# Sleep problems during COVID-19 pandemic and its’ association to psychological distress: A systematic review and meta-analysis

**DOI:** 10.1016/j.eclinm.2021.100916

**Published:** 2021-06-10

**Authors:** Zainab Alimoradi, Anders Broström, Hector W.H. Tsang, Mark D. Griffiths, Shahab Haghayegh, Maurice M. Ohayon, Chung-Ying Lin, Amir H. Pakpour

**Affiliations:** a Social Determinants of Health Research Center, Research Institute for Prevention of Non-Communicable Diseases, Qazvin University of Medical Sciences, Qazvin, Iran; b Department of Nursing, School of Health and Welfare, Jönköping University, Jönköping, Sweden; c Department of Rehabilitation Sciences, The Hong Kong Polytechnic University, Hung Hom, Hong Kong; d International Gaming Research Unit, Psychology Department, Nottingham Trent University, Nottingham, UK; e Department of Biostatistics, Harvard T. H. Chan School of Public Health, Boston, Massachusetts, USA; f Stanford Sleep Epidemiology Research Center (SSERC), School of Medicine, Stanford University, CA, USA; g Institute of Allied Health Sciences, College of Medicine, National Cheng Kung University, Tainan, Taiwan; h Department of Clinical Neurophysiology, Linköping University Hospital, Linköping, Sweden; i Department of Public Health, National Cheng Kung University Hospital, College of Medicine, National Cheng Kung University, Tainan, Taiwan; j Department of Occupational Therapy, College of Medicine, National Cheng Kung University

**Keywords:** COVID-19, Sleep problems, Healthcare workers, COVID-19 patients, General population, Meta-analysis

## Abstract

**Background:**

The emerging novel coronavirus disease 2019 (COVID-19) has become one of the leading cause of deaths worldwide in 2020. The present systematic review and meta-analysis estimated the magnitude of sleep problems during the COVID-19 pandemic and its relationship with psychological distress.

**Methods:**

Five academic databases (*Scopus, PubMed Central, ProQuest, ISI Web of Knowledge,* and *Embase*) were searched. Observational studies including case-control studies and cross-sectional studies were included if relevant data relationships were reported (i.e., sleep assessed utilizing the Pittsburgh Sleep Quality Index or Insomnia Severity Index). All the studies were English, peer-reviewed papers published between December 2019 and February 2021. PROSPERO registration number: CRD42020181644.

**Findings:**

168 cross-sectional, four case-control, and five longitudinal design papers comprising 345,270 participants from 39 countries were identified. The corrected pooled estimated prevalence of sleep problems were 31% among healthcare professionals, 18% among the general population, and 57% among COVID-19 patients (all *p*-values < 0.05). Sleep problems were associated with depression among healthcare professionals, the general population, and COVID-19 patients, with Fisher's Z scores of -0.28, -0.30, and -0.36, respectively. Sleep problems were positively (and moderately) associated with anxiety among healthcare professionals, the general population, and COVID-19 patients, with Fisher's z scores of 0.55, 0.48, and 0.49, respectively.

**Interpretation:**

Sleep problems appear to have been common during the ongoing COVID-19 pandemic. Moreover, sleep problems were found to be associated with higher levels of psychological distress. With the use of effective programs treating sleep problems, psychological distress may be reduced. Vice versa, the use of effective programs treating psychological distress, sleep problems may be reduced.

**Funding:**

The present study received no funding.

**Research in context**

**Evidence before this study**

The novel coronavirus disease 2019 (COVID-19) pandemic has caused psychological problems and sleep problems in different populations, including healthcare professionals, COVID-19 infected individuals, and the general population.

**Added value of this study**

Patients with COVID-19 infection had the highest prevalence of sleep problems, and healthcare professions had the second highest prevalence of sleep problems. Moderate associations between sleep problems and psychological distress (including depression and anxiety) were found.

Patients with COVID-19 infection and health professions are at risk of having sleep problems, and that there are moderate associations between sleep problems and psychological distress.

**Implications of all the available evidence**

These data emphasize the need of programs and treatments to assist different populations in overcoming sleep problems and psychological distress, especially patients with COVID-19 infection and health professions.

Alt-text: Unlabelled box

## 1 Introduction

Prior to 2020, respiratory diseases were the fourth leading cause of death [1]. However, with the outbreak of the novel coronavirus disease 2019 (COVID-19) in December 2019, respiratory infections caused more deaths due to COVID-19 [2]. According to the World Health Organization (WHO) as of April 16, 2021, there were over 137,866,000 known cases of COVID-19 and over 2,965,000 cases of COVID-19 death worldwide [3].

Prior research has found that the prevalence of COVID-19 is associated with major psychological distress and significant symptoms of mental health illness [4], [5], [6], [7], [8]. The sudden onset of a threatening illness puts great pressure on healthcare workers [9]. Consequently, healthcare workers may have impaired sleep because they need to deal with the illness, suffer from the high risk of death, and adapt to irregular work schedules and frequent shifts [10], [11], [12], [13], [14], [15]. They may experience sleep problems, anxiety, depression, and stress when faced with this major public health threat [16], [17], [18]. Due to their job demands, they are in frequent contact with patients and therefore suffer from extremely high-level stress. Therefore, they may develop acute sleep problems, including poor sleep quality and experience too little sleep [19]. Given that healthcare professionals are the frontline workers who take care of patients, their health is extremely important. More specifically, if healthcare providers have any health issues that prevent them from taking care of patients, their local communities more specifically, and their country more generally, will encounter a huge challenge of healthcare burden and consequently impact on all residents’ health.

In addition to healthcare workers, the general population is likely to develop mental health and sleep problems due to the impacts of COVID-19 [20] because a substantial change in lifestyle is a huge stressor [21,22]. For example, individuals may need to self-isolate and quarantine at home, avoid social activities for leisure and recreation that they had participated in previously, and strictly obey the new policies to minimize spread of the virus (e.g., wearing a mask in public areas) [23,24]. The general population may also receive threatening information such as daily statistics concerning COVID-19 infection and deaths reported from the news or social media [25,26]. With the lifestyle changes and threatening information, the general population may avoid contact with other individuals due to great fear of infection, developing feelings of helplessness or suffering from panic [27]. In other words, the general population might experience psychological problems directly due to the COVID-19 pandemic [28].

Different factors contributing to insomnia and psychological problems have been reported. The most important risk factors for insomnia and mental health problems during the COVID-19 pandemic are being a healthcare worker, having an underlying illness, living in rural areas, being a woman, and being at risk of contact with COVID-19 infected patients. Among non-medical healthcare workers, having an underlying disease is a risk factor for insomnia and mental health problems [29]. Indeed, among the natural and non-natural disasters that can occur to humans, the COVID-19 pandemic has caused severe psychological distress due to the large number of individuals affected globally and the contagious and deadly nature of the virus [30]. The COVID-19 pandemic as a worldwide public health issue is a traumatic event that has affected both the sleep and mental health of the general public and healthcare providers [31], [32], [33], [34], [35]. Moreover, several policies implemented to reduce the spread of COVID-19 (e.g., quarantine) have been found to have some negative effects on an individuals’ psychological health [34].

Because sleep is important for human beings to maintain daily functions [36], several studies have focused on sleep problems all with the use of self-report data during the COVID-19 pandemic. Different findings regarding the sleep and psychological problems during COVID-19 in different populations have been reported among these studies. For example, Zhang et al. reported that the prevalence of insomnia was higher among non-medical healthcare workers (e.g., students, community workers, and volunteers) than among medical healthcare workers (prevalence rate of 38.4 vs. 30.5%, *p*<.01). Wang et al. reported higher prevalence of sleep problem among medical staff compared to non-medical staff comprising students, community workers, and volunteers (66.1% vs. 47.8, *p*<.01) and frontline healthcare providers compared to non-frontline medical workers (68.1 vs. 64.5, *p*=0.14) [37].

The quality of sleep during the COVID-19 pandemic and its related factors have been reported in an increasing number of studies. A recent study conducted a meta-analysis to understand the sleep problems during the COVID-19 pandemic [38]. The study found that the pooled prevalence rate of sleep problems globally was 35.7%, with the most affected group being patients with COVID-19 (74.8%), followed by healthcare providers (36.0%), and the general population (32.3%). In addition, sleep difficulties and psychological distress due to COVID-19 on those patients with COVID-19 were reported in a cohort study [39]. Patients with COVID-19 had sleep difficulties, depression, and anxiety at six months after acute infection. Another systematic review found the associations between COVID-19 and psychiatric symptoms among patients with mental illness, healthcare workers, and non-healthcare workers [40]. However, only the information on sleep difficulties has been well analyzed using robust meta-analysis method. Therefore, psychological distress and the associations between sleep problems and psychological distress have yet to be synthesized. Given the significant number of published studies on sleep quality, psychological distress, and related factors, and the importance of systematic reviews and meta-analyses in summarizing and analyzing the results of existing studies, the present study was designed and conducted with the aim of estimating sleep problems during the COVID-19 period (January to October, 2020) and its relationship with psychological distress.

## 2 Methods

The present systematic review was conducted utilizing the Preferred Reporting Items for Systematic Reviews and Meta-Analyses (PRISMA) guidelines [41]. A systematic literature search was carried out utilizing five academic databases, and relevant studies were extracted and their methodological quality was assessed using the Newcastle Ottawa Scale (NOS) checklist. Findings were synthesized using a meta-analysis approach. The protocol was registered in the PROSPERO International prospective register of systematic reviews (ID code: CRD42020181644 [42]).

### 2.1 Search strategy

Five academic databases including *Scopus, PubMed Central, ProQuest, ISI Web of Knowledge,* and *Embase* were searched systematically between February 17 to 19, 2021. The search terms were extracted from published reviews and primary studies in addition to PubMed Medical Subject Headings (MeSH). The main search terms were ‘sleep’ and ‘COVID-19’. The Boolean search method (AND/OR/NOT) was used to develop the search. Search syntax was customized based on the advanced search attributes of each database. The full search strategy for each database is provided in Supplementary Table 1. Additionally, further sources (i.e., reference lists of included studies and systematic reviews of published papers) were searched to increase the likelihood of retrieving relevant empirical studies.

### 2.2 Inclusion criteria

Observational studies including case-control studies and cross-sectional studies were included if relevant data relationships were reported (i.e., sleep assessed using the Pittsburgh Sleep Quality Index or Insomnia Severity Index). More specifically, if the studies were included if they estimated the prevalence of sleep disorders and/or examined the relationship between sleep and psychological distress using Pearson's correlation coefficient (e.g., if the odds ratio [OR] information reported by the studies could be converted into Pearson's correlation coefficient; detailed information in *2.6 Data synthesis*). English, peer-reviewed papers published between December 2019 and August 2020 were included. There were no limitations regarding participants’ characteristics.

#### 2.2.1 Primary outcome

Estimation of sleep problems frequency was the primary outcome. Sleep problems were defined in a broad category of sleep disorders characterized by either hypersomnolence or insomnia. The three major subcategories of sleep problems were intrinsic (i.e., arising from within the body), extrinsic (secondary to environmental or pathological conditions), and disturbances of circadian rhythm. Sleep problems had to have been assessed using valid and reliable psychometric scales or confirmed with defined cut-off points for characterizing as sleep problems. More specifically, Pittsburgh Sleep Quality Index (PSQI) and Insomnia Severity Index (ISI) were used to assess the primary outcomes because PSQI and ISI have items assessing the three major subcategories of the aforementioned sleep problems. For instance, a global score of 5 or more indicates poor sleep quality on the Pittsburgh Sleep Quality Index [43], or total score of 8 or more on the Insomnia Severity Index [44]

#### 2.2.2 Secondary outcomes

There were three secondary outcomes: (i) association of sleep problems with psychological distress in the context of the COVID-19 pandemic; (ii) heterogeneity and its possible sources; and (iii) moderator variables in association of sleep problems and psychological distress related to COVID-19 pandemic. Ridner defined psychological distress (PD) as: *“a state in response to stressors marked by perceived discomfort and inability to cope”* [45]. In the present study, psychological distress was considered as either depression (defined as having depressed mood) and/or anxiety (defined as having excessive worry and being nervous). These had to have been assessed using valid and reliable psychometric scales. That is, studies were excluded if psychological distress was assessed using a non-psychometrically validated self-designed questionnaire. Moreover, in the present systematic review and meta-analysis, depression, and anxiety were treated as continuous variables.

### 2.3 Study screening and selection

In the first step, title and abstract of all retrieved papers were screened independently by two researchers based on the inclusion criteria. The full texts of potentially relevant studies were further examined based on the aforementioned criteria. In this process, relevant studies were selected.

### 2.4 Quality assessment

The Newcastle Ottawa Scale (NOS) was used to evaluate the methodological quality of the studies in observational studies. Three characteristics (i.e., selection, comparability, and outcome) were examined with the NOS checklist. The checklist has three versions for evaluating cross-sectional studies (seven items), case-control studies (eight items), and cohort studies (eight items). Despite a slight difference in number and content of items, each item is rated with a star, except comparability which can have two stars. This results in a maximum quality score of 9 for each study. Studies with less than 5 points are classified as having a high risk of bias [46]. No studies were excluded based on the quality rating. However, subgroup analysis was conducted to assess the impact of quality on pooled effect size

### 2.5 Data extraction

A pre-designed form was prepared to extract data from included studies. Data including first author's name, collection date, study design, country, number of participants, gender, mean age, scales used to assess psychological distress and sleep problems, numerical results regarding the frequency of sleep problems, and relationship between sleep problems and psychological distress. It should also be noted that study selection, quality assessment, and data extraction were processes performed independently by two reviewers. Disagreements were resolved through discussion.

### 2.6 Data synthesis

A quantitative synthesis using STATA software version 14 was conducted. Meta-analysis was run using random effect model because included studies were taken from different populations, and both within-study and between-study variances should be accounted for [47]. The Q Cochrane statistic was used to assess heterogeneity. Also, the severity of heterogeneity was estimated using the I^2^ index. Heterogeneity is interpreted as (i) mild when I^2^ is less than 25%, (ii) moderate when I^2^ is 25 to 50%, (iii) severe when I^2^ is 50 to 75%, and (iv) highly severe when I^2^ is greater than 75% [48].

Two key measures were selected for present study: (i) prevalence of sleep problems and (ii) correlation of sleep problem with psychological distress. The numerical findings regarding prevalence of sleep problems were reported consistently in 177 included studies. This key measure and its 95% confidence interval (CI) are reported. However, the association between sleep problems and psychological distress was reported differently in the included studies. Pearson's correlation coefficient was the selected effect size for meta-analysis. Due to the inconsistency in reporting numerical findings of this association, the other effect sizes of standardized mean difference and crude odds ratio were transformed into Pearson's correlation coefficients [49,50] using the *Psychometrica* website [51]. Also, Pearson's *r* correlation coefficient was converted to Fisher's z, due to the potential instability of variance. Consequently, all analyses were performed using Fisher's z values as effect size (ES) [52,53]. Fisher's z-transformation was applied using the following formula: z = 0.5 × ln(1+r-1-r). The standard error of z was calculated based on the following formula: SEz = 1/√ (n-3) [54]. Therefore, the selected measure of effect, selected for current meta-analysis, is expressed as Fisher's z score and its 95% CI.

For assessing moderator analysis and finding the possible sources of heterogeneity, subgroup analysis or meta-regression was carried out based on the number of studies in each group. Moreover, the three subgroups for synthesized analyses (i.e., general population, healthcare professionals, and patients) did not have any overlapping participants. More specifically, the general population did not include healthcare professionals or patients. If the number of studies in any group was less than four studies, meta-regression was used. Funnel plot and the Begg's Test were used to assess publication bias [55]. The Jackknife method was used for sensitivity analysis [56].

### 2.7 Role of the funding source

The present systematic review and meta-analysis did not receive any specific funding. However, one of the authors (Dr. C-Y Lin) received a grant on COVID-19 research to support his works on COVID-19. The grant that Dr. Lin received had no role in study design, data collection and analysis, decision to publish, or preparation of the manuscript.

## 3 Results

### 3.1 Study screening and selection process

**Figure 1:**
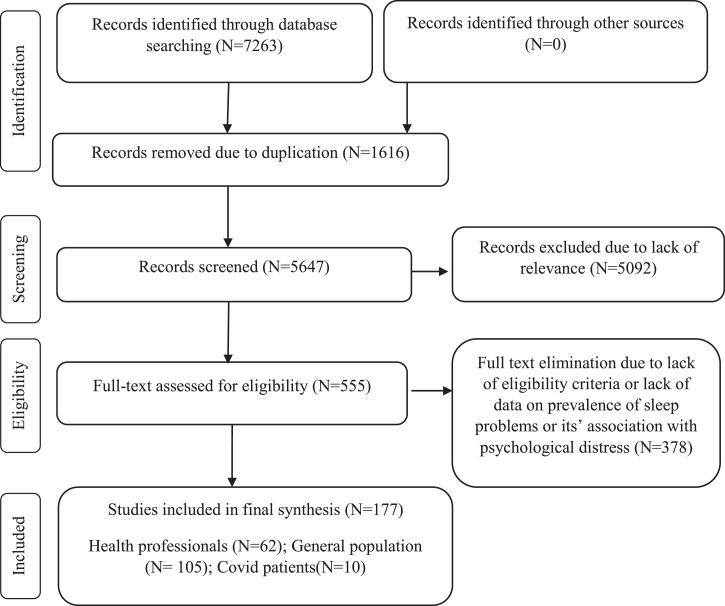
PRISMA Flowchart of selected studies

The initial search in five databases resulted in 7263 studies: *Scopus* (n=2518), *ISI Web of Knowledge* (n=474), *PubMed* (n=338), *Embase* (n=1426), and *ProQuest* (n=2507). After removing duplicate papers, a further 5647 papers were screened based on title and abstract. Finally, 555 papers appeared to be potentially eligible and their full-texts were reviewed. In this process, 177 studies met the eligibility criteria and were pooled in the meta-analysis. Figure 1 shows the search process based on the PRISMA flowchart.

### 3.2 Study description

All the included studies (N=177) collected the data online and comprised 345,270 participants from 39 different countries (, Algeria, Argentine, Australia, Austria, Bahrain, Bangladesh, Belgium, Brazil, Canada, China, Colombia, Egypt, Ethiopia, Finland, France, Greece, India, Iran, Iraq, Israel, Italy, Lebanon, Malaysia, Morocco, Nepal, Netherlands, Nigeria, Oman, Pakistan, Palestine, Poland, Qatar, Saudi Arabia, Serbia, Spain, Sweden, Syria, Turkey, Tunisia, United Arab Emirates, UK, USA, and Vietnam). Of these, 28 studies collected data during the national lockdown period in the respective countries. The two countries with the highest number of eligible studies were China (N=76) and Italy (n=17). The smallest sample size was 20, and the largest sample size was 56,932. The mean age of participants varied from 15.26 years to 69.85 years. Approximately two-thirds of overall participants were females (63.5%) and one-third were married (35.33%). The most frequently used study design was cross-sectional (n=168). Four studies had a case-control design and five studies had a longitudinal design. In longitudinal studies, collected data during the COVID-19 pandemic were extracted. Various measures were used to assess sleep problems, with the Insomnia Severity Scale (ISI; n=93) and Pittsburgh Sleep Quality Index (PSQI; n=60) being the most frequently used scales in the studies. Psychological distress was also assessed with different measures, with the Patient Health Questionnaire (PHQ; n=73) and Generalized Anxiety Disorder Scale (GAD; n=75) being the most frequently used scales in the studies. Table 1 provides the summary characteristics of all included studies.

**Table 1:** Data extraction- Summarized characteristics

ID	Authors	Year	Country	Collection Date	Lock down Period	Design	Participant Group	Sample Size	Sex % Female	% Married	Mean Age/ Age range (Years)	NOS	Sleep Problem Scale	Psychological Distress Scale
2	Xiao [67]	2020	China	January and February 2020	no	Cross-sectional	Medical Staff	180	71.7	67.8	32.31	5	PSQI	Self-Rating Anxiety Scale
3	Zhang [68]	2020	China	29 January to 3 February 2020	no	Cross-sectional	Medical staff	1563	82.73	63.92	18 to above 60	5	ISI	GAD-7 PHQ-9
5	Huang [69]	2020	China	3 February to 10 February 2020	no	Cross-sectional	Volunteer population	603	69		36.5	5	PSQI	GAD-7 & CESD
10	Xiao [70]	2020	China	January 2020	yes	Cross-sectional	Individuals who self-isolated	170	40.5	64.7	37.78	4	PSQI	Self-Rating Anxiety Scale
12	Zhang [29]	2020	China	February 19 to March 6, 2020	no	Cross-sectional	Medical health workers	2182	64.2	82	less than 18 to above 60	5	ISI	PHQ-4
16	Wanqiu [71]	2020	China	24 Feb to 25 Feb 2020	no	Cross-sectional	Workforce	673	25.6	54.4	30.8	5	ISI	Impact of Event Scale-Revised, DASS-21
18	Qi [32]	2020	China	February 2020	no	Cross-sectional	Frontline medical workers	1306	80.4	68.4	33.1	3	PSQI	anxiety and depression VAS
21	Rossi [72]	2020	Italy	March 27th and April 6th 2020	no	Cross-sectional	General population	18147	79.5		38	5	ISI	PHQ-9 GAD-7
23	Tu [73]	2020	China	February 7 to 25, 2020	no	Cross-sectional	Frontline nurses	100	100	70	34.44	7	PSQI	PHQ-9 GAD-7
24	Jahrami [74]	2020	Bahrain	April 2020	no	Cross-sectional	Frontline healthcare workers	257	70	89.1	40.2	7	PSQI	PSS (Perceived Stress Scale)
25	Lin [31]	2020	China	February 5 to 23, 2020	no	Cross-sectional	Adults	5461	70.1		less than 18 to above 60	3	ISI	PHQ-9 GAD-7
26	Magnavita [75]	2020	Italy	March 2020	no	Cross-sectional	Health care workers	595	70.1	76.13	less than 35 to above 55	7	Sleep Condition Indicator (SCI)	Goldberg Anxiety and Depression Scale (GADS)
27	Romero-Blanco [76]	2020	Spain	1 and 15 April, 2020	yes	Cross-sectional	Nursing students/ post 4 weeks lockdown	207	81.6		20.57	6	PSQI	EQ-5D
28	Fu [77]	2020	China	February 18 to 28, 2020	no	Cross-sectional	Wuhan residents	1242	69.73	33.7	above 18	5	AIS	PHQ-9
29	Guo [78]	2020	China	1–10 February 2020	no	Cross-sectional	Adults	2441	52.4	70.3	18 to above 51	6	PSQI	CESD
30	Zhang [79]	2020	China	February 19 to March 20, 2020	no	Longitudinal surveys	College students	66	62.12		20.70	5	PSQI	DASS-21
32	Li [80]	2020	China	25 April to 9 May 2020	no	Cross-sectional	Workers with income losses	398	49.5	49.5	18 to above 40	9	ISI	GAD-7 PHQ-9
34	Wang [81]	2020	China	30 January to 7 February 2020	no	Cross-sectional	Medical workers	123	90	30.08	33.75	6	PSQI	SAS SDS
35	Hu [82]	2020	China	March 7 to 24, 2020	no	Cross-sectional	COVID-19 inpatients	85	49.4	85.9	48.8	6	ISI	GAD-7 PHQ-9
36	Yang [83]	2020	China	March 5 to 14, 2020	no	Cross-sectional	General population	2,410	49.2	76.55	36.3	5	PSQI	GAD-7 PHQ-9
37	Wang [68]	2020	China	26 February and 3 March, 2020	no	Cross-sectional	Medical staff	274	77.4	81.8	37	5	PSQI	GAD-7 PHQ-9
39	Marelli [84]	2020	Italy	March 24 to May 3, 2020	no	Cross-sectional	University students and staff	400	75.8		29.93	5	PSQI	Beck Anxiety Inventory/ Beck Depression Inventory- II
42	Wu [85]	2020	China	February 2020	no	Case- control	Frontline vs. non frontline clinical staff	120	74.15		33.65	4	PSQI	Self-rating Anxiety Scale (SAS), Selfrating Depression Scale (SDS)
45	Gualano [86]	2020	Italy	April 19th and May 3rd 2020	yes	Cross-sectional	General population	1515	65.6	61.1	42	5	ISI	GAD-7 PHQ-9
53	Peng [87]	2020	China	February 14 to March 4, 2020	yes	Cross-sectional	General population	2237	41.66	68.44	35.93	5	PSQI	Zung's Self-Rating Depression Scale (SDS) & self-rating anxiety scale
57	Pieh [88]	2020	Austria	April 15th to 30th, 2020	yes	Cross-sectional	General population	1005	52.7		18 to above 65	6	ISI	GAD-7 PHQ-9
59	Zhao [89]	2020	China	February 18 to 25, 2020	no	Cross-sectional	General population	1630			29.17	5	PSQI	Self-Rating Anxiety Scale
61	Huang [90]	2020	China	February 3 to 17, 2020	no	Cross-sectional	General public	7236	54.6		35.3	4	PSQI	GAD-7 CES_D
63	Assenza [91]	2020	Italy	April 11, 2020	no	Cross-sectional	General population	928	74.46	41.81	40.10	5	PSQI	Beck Depression Inventory- II
64	Que [92]	2020	China	February 2020	no	Cross-sectional	Healthcare workers	2285	69.06		31.06	5	ISI	GAD-7 PHQ-9
65	Zhuo [67]	2020	China	March 2020	no	Cross-sectional	Medical staff	26	46.15		41.92	5	ISI	Chinese version of the Self-Reporting Questionnaire (SRQ-20)
67	Mazza [93]	2020	Italy	April 6 to June 9, 2020	no	Cross-sectional	COVID-19 survivors	402	65.92		57.8	6	Medical Outcomes Study Sleep Scale (MOS-SS)	Zung Self-Rating Depression Scale/ 13-item Beck's Depression Inventory (BDI-13) /State-Trait Anxiety Inventory form Y (STAI-Y)
68	Song [94]	2020	China	9–22 April, 2020	no	Cross-sectional	People resuming Work	709	74.2		35.35	5	ISI	GAD-7 CESD
69	Wang [95]	2020	China	2nd and 3rd February 2020	no	Cross-sectional	Medical staff	1045	85.8			7	ISI	HADS
70	Shi [96]	2020	China	February 28 to March 11, 2020	no	Cross-sectional	General population	56932	52.1	77.2	35.97	7	ISI	GAD PHQ
71	Hao [97]	2020	China	19 to 22 February 2020	yes	Case control	Psychiatric patients (n = 76); Healthy controls (n = 109)	185	49.75		32.95	4	ISI	DASS-21
72	Caballero-Domínguez [98]	2020	Colombia	March 30 to April 8, 2020	yes	Cross-sectional		700	68.0	48	37.1	6	AIS	WHO-5 (depression) CESD
73	Liu [99]	2020	USA	April 13 to May 19, 2020	no	Cross-sectional	Young adults with suspected and reported psychiatric diagnoses	898	81.3		24.47	5	MOS-Sleep Problems	PHQ-8 GAD-7
74	Stojanov [100]	2020	Serbia		no	Cross-sectional	Healthcare professionals	201	65.95		40.8	3	PSQI	GAD-7, Self-rating Depression Scale
76	Cheng [101]	2020	China	February 9th to the 13th, 2020	no	Cross-sectional	Medical staff	534	82.4		20 to above 50	6	PSQI	self-rating anxiety scale
77	Cellini [102]	2020	Italy	March 24 to 28, 2020	yes	Cross-sectional	COVID-19 lockdown	1310	67.18		23.91	3	PSQI	DASS-21
78	Amerio [103]	2020	Italy	March 15 to April 15, 2020	no	Cross-sectional	General practitioners	131	48.1	70.2	52.31	3	ISI	PHQ-9 GAD-7
79	Cai [104]	2020	China	February 11 to 26, 2020	no	Case-control	Frontline and non-frontline medical workers	2346	70	83.2	30.55	5	ISI	Beck Anxiety Inventory PHQ-9
82	Wang [37]	2020	China	March 4 to 9, 2020	no	Cross-sectional	Healthcare workers	2737	64.5	70.9	18-65	6	PSQI	HADS
85	Idrissi [105]	2020	Morocco	April 1, to May 1, 2020	yes	Cross-sectional	General population	846	52.2		35.9	5	AIS, ESS	Hamilton Anxiety Rating Scale (HARS) and Beck Depression Inventory (BDI
87	Zhou [106]	2020	China	March 8 to March 15, 2020	no	Cross-sectional	Adolescents and young adults	11835	57.7		17.41	6	PSQI	GAD-7 PHQ-9
96	Juanjuan [107]	2020	China	February 16 to 19, 2020	no	Cross-sectional	Breast cancer patients	658	100		less than 45 to above 65	6	ISI	GAD-7 PHQ-9
97	Huang [108]	2020	China	February 2 and March 5, 2020	yes	Cross-sectional	Patients with epilepsy	362	45.86		10 to above 60	7	ISI	GAD-7 PHQ-9
98	Mamun [63]	2020	Bangladesh	April 1-10, 2020	no	Cross-sectional	General population	10067	28.2	43.9	29.94	6	ISI	PHQ-9
11	Lai [109]	2020	China	January 29 to February 3, 2020	no	Cross-sectional	Healthcare workers	1257	76.7	66.7	18 to above 40	6	ISI	GAD-7 PHQ-9
13	Kang [110]	2020	China	January 29 to February 4, 2020	no	Cross-sectional	Healthcare workers	994	85.5	56.9	18 to above 50	6	ISI	GAD-7 PHQ-9
38	Zhan [111]	2020	China	March 3–10, 2020	no	Cross-sectional	Healthcare workers	1794	97		less than 25 to above 65	6	AIS	
43	Wang [112]	2020	China	23 March to 26 April 2020	yes	Cross-sectional	General population	2289	51.38	30	27.5	6	PSQI	
46	Zhou [113]	2020	China	24 March to 3 April 2020	no	Cross-sectional	Healthcare workers	1931	95.4	63.4	35.08	5	PSQI	
56	Zhang [114]	2020	China	January 25 and March 15	no	Retrospective cohort	Covid-19 patients	136	42.2	95.6	63	6	PSQI	
554	Wasim [115]	2020	Pakistan	20th May to 3rd June 2020	no	Cross-sectional	Tertiary care hospital dealing with corona patients	356	52.00	51.40	20 to above 50	6	ISI	DASS-21
553	Lu [116]	2020	China	May 13 to 20	no	Cross-sectional	Middle school students	965	42.40		15.26	9	Youth Self-Rating Insomnia Scales	PHQ-9 GAD-7
544	Yitayih [117]	2020	Ethiopia	22 and 28 March 2020	no	Cross-sectional	Healthcare professionals	249	52.60		27.40	6	ISI	0.00
542	Tselebis [118]	2020	Greece	half of May 2020	no	Cross-sectional	Nursing Staff	150	80.00		42.29	7	AIS	0.00
541	Liu [119]	2021	China	7 to 17 March 2020	no	Cross-sectional	Obstetrics staff	2259	97.70		16–65	5	ISI	PHQ-9 GAD-7
540	Rossi [120]	2020	Italy	March 25th and April 7th. 2020	no	Cross-sectional	General population + healthcare professionals	24048	80.39		48.31	6	ISI	PHQ-9 GAD-7
537	Sharma [121]	2020	India	0	no	Cross-sectional	Obstetrics staff	184	58.70	54.35	20 to above 50	5	ISI	DASS-21
536	Ammar [122]	2020	Multi country	April 11 to, 2020	Data on both before and during lockdown period is provided	Cross-sectional	General population	1047	53.80	53.70	18 to above 50	6	PSQI	0.00
535	Tiete [123]	2021	Belgium	April 17th to May 25th, 2020	no	Cross-sectional	Healthcare professionals	647	78.40	80.50	20 to above 50	8	ISI	DASS-21
511	Franceschini [124]	2020	Italy	March 10 to May 4, 2020	yes	Cross-sectional	General population	6439	73.10	65.10	33.90	6	Medical Outcomes Study–Sleep Scalbe (MOS-SS	DASS-21
507	Huang [125]	2020	China	0	no	Cross-sectional	Nurses	881	91.20			5	PSQI	0.00
506	Elkholy [126]	2020	Egypt	April and May 2020	no	Cross-sectional	Healthcare workers	502	50.00	60	20 to above 40	8	ISI	PHQ-9 GAD-7
502	Yang [127]	2020	China	6 to 8 June 2020	no	Cross-sectional	Healthcare workers	15000	57.10		less than 18 to above 60	6	ISI	PHQ-9
495	Yang [128]	2020	China	January to May 2020	no	Cross-sectional	Young cancer patients	197	54.82		36.50	5	PSQI	self-rating Anxiety Scale
490	Caballero‐Domínguez [129]	2020	Colombia	March 30 to April 8, 2020	yes	Cross-sectional	General population	700	68	48	37.10	8	AIS	Well‐Being Index
462	Khamis [130]	2020	Oman	first two weeks of April 2020	no	Cross-sectional	Healthcare professionals	402	100	77.30	36.40	5	SQS	GAD-7
472	Sañudo [131]	2020	Spain	one-week period from February 2020 & 24 March to 3 April 2020 in locking period	data on both prior and during locking period	Cross-sectional	General population	20	47		22.60	5	PSQI	
460	Jain [132]	2020	India	12 to 22 May 2020	no	Cross-sectional	Anesthesiologists	512	44.30	64.70	less than 30 to above 60	7	ISI	GAD-7
454	Agberotimi [133]	2020	Nigeria	March 20 to April 19, 2020	yes	Cross-sectional	General population + healthcare professionals	884	45.50	65.30		6	ISI	PHQ-9 GAD-7
447	Bhat [134]	2020	Kashmir	4 to 10 April 2020	no	Cross-sectional	General population	264	27.70		less than 18 to above 60	8	PSQI	HADS
442	McCracken [135]	2021	Sweden	14th of May and the June 11, 2020	no	Cross-sectional	General population	1102	75.20	56.30	36.90	6	ISI	PHQ-9 GAD-7
439	Trabelsi [136]	2021	Multi country	6 April to 28 June 2020	data on both prior and during locking period	Cross-sectional	General population	5056	59.40	50.20	less than 18 to above 55	6	PSQI	
438	Chi [137]	2020	China	May 13 and 20, 2020	no	Cross-sectional	Adolescents	1794	43.90		15.26	7	YSIS	PHQ-9 GAD-7
420	Liu [138]	2021	China	February 1 to 10th in 2020	no	Cross-sectional	General population	2858	53.60	60.20	less than 18 to above 50	6	PSQI	
410	Alamrawy [139]	2021	Egypt	2 July to 23 July 2020	no	Cross-sectional	Young adults of both genders aged between 14 and 24 years	447	70.20		20.72	8	ISI	PHQ-9 GAD-7
408	Haravuori [140]	2020	Finland	4 June to 26 June 2020	no	Cross-sectional	General population + healthcare professionals	4804	87.50		45	6	ISI	PHQ-2 Overall Anxiety and Impairment Scale (OASIS)
405	Khaled [141]	2021	Qatar	Feb-20	no	Cross-sectional	General population	1160	53.20	79.30	above 18	8	Sleep Condition Indicator (SCI)	PHQ-9 GAD-7
403	Alomayri [142]	2020	Saudi Arabia	July and August 2020	no	Cross-sectional	Patients with atopic dermatitis	400	86		18 to above 55	7	PSQI	0.00
397	Akıncı [143]	2021	Turkey	April and May of 2020	no	Cross-sectional	Patients hospitalised with COVID-19	189	41	82.50	46.27	6	PSQI	HADS
394	Barua [144]	2021	Bangladesh	1st April to 30th May 2020	no	Cross-sectional	Healthcare professionals	370	39.70	66.80	30.50	8	Sleep Condition Indicator (SCI-02)	PHQ-2 GAD-2
391	Wang [145]	2020	China	February 3 to 7, 2020	no	Cross-sectional	General population	19372	51.96		11 or older	6	ISI	PHQ-9 GAD-7
389	Fidanci [146]	2020	Turkey	May-20	no	Cross-sectional	Healthcare professionals	153	67.30		33.40	5	PSQI	0.00
382	Chouchou [147]	2020	France	0	data on both prior and during locking period	Cross-sectional	General population	400	58.25		29.80	6	PSQI	0.00
378	Cheng [148]	2020	UK & US	16 - 22 March 2020 & 18–24 May 2020	no	Cross-sectional	General population	2278	53.5			6	PROMIS	State-Trait Anxiety Inventory
376	Gu [87]	2020	China	February 15 -22, 2020	no	Cross-sectional	Patients with COVID-19	461	64.90	95.90	18 to above 50	5	ISI	PHQ-9 GAD-7
371	Pedrozo-Pupo [149]	2020	Colombia	0	no	Cross-sectional	Asthma and COPD patient	227	64.70		60.40	5	AIS	PHQ-9
370	Targa [150]	2020	Spain	April 28 to May 12, 2020	no	Cross-sectional	General population	71	75.00		40.70	5	PSQI	Profile of mood states- depression
364	Than [151]	2020	Vietnam	March and April 2020	no	Cross-sectional	Healthcare professionals	173	68.20		31.00	5	ISI	DASS-21
359	Youssef [152]	2020	Egypt	Apr-20	no	Cross-sectional	Healthcare professionals	540	45.60	74.10	37.30	6	ISI	DASS-21
357	Ge [153]	2020	China	February 10th to 13th, 2020	no	Cross-sectional	Undergraduate student	2009	50.97			6	ISI	GAD-7
348	Almater [154]	2020	Saudi Arabia	March 28 to April 4 2020	no	Cross-sectional	Ophthalmologists	107	43.90		32.90	8	ISI	GAD-7
315	Gupta [155]	2020	India	early May 2020	no	Cross-sectional	General population + healthcare professionals	958	41	67	37	6	ISI	0
4	Varma [156]	2021	Australia	April 9 and May 25, 2020	yes	Cross-sectional	General population	1653	67.70		42.90	6	PSQI	PHQ-9 State-Trait Anxiety Inventory
5	Li [157]	2021	China	May 22 and July 15, 2020	no	Cross-sectional	Clinically stable older patients with psychiatric disorders	1063	67.40	90.40	62.80	8	ISI	PHQ-9 GAD-7
6	Duran [158]	2021	Turkey	Oct-2020	no	Cross-sectional	General population	405	70.86	36.30		8	PSQI	
7	Yang [159]	2021	China	March 5 -9, 2020	no	Cross-sectional	Healthcare providers	1036	72.90	66.00	20 to above 50	8	ISI	
8	Martínez-de-Quel- Before Lock down [160]	2021	Spain	March 16 and March 31, 2020 & April 30 and May 11, 2020	data on both prior and during locking period	Longitudinal	General population	161	37.00		35.00	6	PSQI	
12	Khoury [161]	2021	Canada	June 3 and July 31, 2020	no	Cross-sectional	Pregnant individuals	303	100.00	100.00	32.13	7	ISI	CESD Cambridge Worry Scale (CWS)
17	Wang [162]	2021	China	January 28 to March 31, 2020	no	Cross-sectional	General population	5676	71.40	68.90		6	ISI	PHQ-9 GAD-7
25	Zreik [163]	2021	Israel	20 to 30April 2020	yes	Cross-sectional	General population	264	100	100	33.97	5	ISI	Trait Anxiety Scale
38	Zhang [164]	2021	China	mid-February to late March 2020	no	Cross-sectional	Medical Staff	319	62.1		30.42	7	PSQI	HADS
41	Al Ammari [165]	2021	Saudi Arabia	27 April to 4 May 2020	no	Cross-sectional	Medical Staff	720	64.17	35.14	18 to above 40	6	ISI	PHQ-9 GAD-7
45	Essangri [166]	2021	Morocco	April 8 to April 18, 2020	no	Cross-sectional	Medical Students	549	74	18.4	22	8	ISI	PHQ-9 GAD-7
46	Yitayih [167]	2020	Ethiopia	22 to 28 March 2020	no	Cross-sectional	General population	247	23.5	63.2	30.47	7	ISI	0
47	Xie [168]	2020	China	0	no	Cross-sectional	Pregnant individuals	689	100	100	29.03	6	PSQI	0
48	Zhang [169]	2021	China	January to February 2020	no	Cross-sectional	Pregnant individuals	456	100	100		6	PSQI	0
57	Massicotte [170]	2021	Canada	28 April and 29 May 2020	no	Cross-sectional	Breast Cancer Patients	36	100	66.7	53.6	5	ISI	HADS
64	Poyraz [171]	2021	Istanbul	March 16 and June 14, 2020	no	Cross-sectional	Covid patient after initial treatment	284	49.8	65	39.7	5	ISI	HADS
67	Chen [172]	2021	China	March 14- 21, 2020	no	Cross-sectional	Breast cancer patients	834	100	86		5	ISI	PHQ-9 GAD-7
69	Lahiri [173]	2021	India	April 20 e May 19, 2020	yes	Cross-sectional	General population	1081	41.72	52.91		8	ISI	GAD-7
70	Cellini [174]	2021	Italy & Belgium	April 1st to May 19th, 2020	Data on both prior and during locking period	Cross-sectional	General population	2272	75.25		38.55	6	PSQI	
75	Lin [119]	2021	Hong Kong	20 February to 29 February 2020	no	Cross-sectional	General population	1897	43.6		36.6	7	PSQI	0
80	Sunil [175]	2021	India	June to july 2020	no	Cross-sectional	Medical staff	313	64.5		Above 21	8	ISI	PHQ GAD
81	Yadav [176]	2021	India	June to August 2020	no	Cross-sectional	COVID-19 patients	100	27		42.9	5	ISI	PHQ GAD
82	Scotta [177]	2020	Argentina	0	yes	Cross-sectional	University students	584	81	42	22.49	6	ISI	0
84	He [178]	2020	China	29 February 2020 to 1 May 2020	no	Cross-sectional	General population, healthcare workers and quarantined population	2689	70.1	42.84	56.84	6	PSQI	PHQ GAD
85	Zhang [179]	2020	China	February 16th to 2020 March 2th.	no	Cross-sectional	Medical staff	524	74.4	80	34.87	6	ISI	PHQ GAD
87	Demartini [180]	2020	Italy	24 to 31 March 2020	no	Cross-sectional	General population + healthcare professionals	432	72		35.9	6	PSQI	DASS-21
91	Cui [181]	2020	China	February 1 to 19, 2020	no	Cross-sectional	Breast cancer patients and female nurses	891	100	74.21	18 to above 40	9	ISI	PHQ GAD
92	Bacaro [182]	2020	Italy	1st of April to 4th May 2020	yes	Cross-sectional	General population	1989	76.17		38.4	7	ISI	HADS
93	Gu [183]	2020	China	February 21 to28, 2020	no	Cross-sectional	Healthcare workers	522	77.6	62.1	18 to above 40	9	ISI	PHQ GAD
95	Liu [184]	2020	China	February 14 to March 29, 2020	no	Cross-sectional	Healthcare workers	606	81.2	74.91	35.77	9	ISI	0
96	Wang [185]	2020	China	February10-20, 2020	no	Cross-sectional	General population	4191	62	81.63	36.15	9	ISI	PHQ BAI
106	Zhou [80]	2020	China	February 28–March 12, 2020	no	Cross-sectional	General population of pregnant and non-pregnant women	859	100	93.25	33.25	9	ISI	PHQ GAD
109	Abdulah [186]	2020	Iraq	0	no	Cross-sectional	Healthcare workers	268	29.9		35.06	8	Athens Insomnia Scale	0
112	Zhou [106]	2020	China	February 14 to March 29, 2020.	no	Cross-sectional	General population + healthcare professionals	1705	73.61	50.85	32.5	9	ISI	PHQ GAD
113	Ren [95]	2020	China	February 14 to March 29, 2020	no	Cross-sectional	General population	1172	69.3	39.3	22	7	ISI	PHQ GAD
114	Cai [187]	2020	China	January 29 to February 2 & February 26 to February 28, 2020	no	Cross-sectional	Nurses	1330	97	56.32	18 to above 40	9	ISI	PHQ GAD
116	Giardino [82]	2020	Argentina	Jun-20	no	Cross-sectional	healthcare workers	1059	72.7		41.7	7	ISI	0
118	Kocevska [188]	2020	Netherlands	0	yes	Cross-sectional	General population	667				7	ISI	0
119	Zhang [189]	2020	China	February 5, 2020, to March 6, 2020	no	Cross-sectional	COVID-19 patients	30	50	80	42.5	9	ISI	PHQ GAD
120	Fazeli [190]	2020	Iran	2 May to 26 August 2020	no	Cross-sectional	Adolescents	1512	43.6		15.51	9	ISI	DASS-21
123	Bajaj [191]	2020	India	25th March 2020-1st April 2020	yes	Cross-sectional	General population	391	53.45		18 to above 40	7	ISI	0
125	Kilani [192]	2020	Arab Countries	17th–24th, April 2020.	no	Cross-sectional	General population	1723	46.78	55	34.9	9	PSQI	0
126	Necho [193]	2020	Ethiopia	July 15 to 30/2020	no	Cross-sectional	individuals living with disabilities	423	40.7	51.4	36.66	9	ISI	PHQ GAD
130	Şahin [194]	2020	Turkey	23 April and 23 May 2020	no	Cross-sectional	Healthcare workers	939	66	65.7	18 to above 40	9	ISI	PHQ GAD
136	McCall [195]	2020	USA	15-May-20	no	Cross-sectional	health care workers	573	72		43.4	9	RDC definition of insomnia disorder	PHQ GAD
137	Lai [196]	2020	UK	April 28 through May 12, 2020	no	Cross-sectional	International university students	124	63.7			9	ISI	PHQ
138	Wang [197]	2020	China	February 21 to March 7, 2020	no	Cross-sectional	College students	3092	66.4			9	Self-Rating Scale of Sleep (SRSS	GAD
139	Sagherian [198]	2020	USA	May–June 2020	no	Cross-sectional	Nursing staff	564	94.06	69.36	18 to above 40	9	ISI	0
150	Magnavita [199]	2020	Italy	27 April and 27 May 2020	no	Cross-sectional	Anesthetists	90	52.2	66.7		9	Sleep Condition Indicator	Goldberg Anxiety and Depression Scale
155	Casagrande [200]	2020	Italy	March 18th to April 2nd, 2020	no	Cross-sectional	General population	2291	74.6		above 18	9	PSQI	GAD
158	Marroquín- sample 2 [201]	2020	USA	March 2020 sample	no	Cross-sectional	General population	435	46.4		39.2	9	ISI	CESD GAD
159	Wang [202]	2020	China	Mar-20	no	Cross-sectional	COVID-19 inpatients	484	50.2	91.7	52.5	9	ISI	PHQ GAD
161	Herrero San Martin [203]	2020	Spain	March 1st to April 30th 2020	no	Cross-sectional	Healthcare workers	170	58.82		36.4	9	PSQI	0
162	Florin [204]	2020	France	April 10 to April 19,2020	yes	Cross-sectional	Healthcare workers	1515	44.3	82.8	45.2	9	ISI	HADS
163	Zhang [205]	2020	China	March 2 to 8, 2020	no	Cross-sectional	General population	3237	47.1	62.7	18 to above 64	9	ISI	PHQ GAD
164	Xia [206]	2020	China	April 20 to 30, 2020	no	Case- control	Patients with Parkinson's disease	288	51.85		60.50	9	PSQI	HADS
165	Zanghì [207]	2020	Italy	4 May to 22 May 2020	no	Cross-sectional	Multiple sclerosis patients	432	64.1	70.3	40.4	9	ISI	0
169	Saracoglu [208]	2020	Turkey	0	no	Cross-sectional	Healthcare workers	220	27.9		29	9	PSQI	PHQ
174	Alnofaiey [209]	2020	Saudi Arabia	May 2020 to August 2020	no	Cross-sectional	Healthcare workers	340	49.1		20-60	9	PSQI	0
176	Saraswathi- During COVID-19 data [210]	2020	India	0	no	Longitudinal study	Medical students in a COVID-19 treating	217	64		20	9	PSQI	DASS-21
179	Badellino [211]	2020	Argentine	March 29 to April 12, 2020	no	Cross-sectional	General population	1985	75.9		36.83	9	ISI	PHQ GAD
181	Bigalke [212]	2020	USA	April 25 and May 18, 2020	Yes	Cross-sectional	General population	103	59		38	6	PSQI	0
182	Alshekaili [213]	2020	Oman	8-17 April 2020	no	Cross-sectional	Healthcare workers	1139	80	86.9	36.3	9	ISI	DASS-21
190	Juanjuan [214]	2020	China	February 16-19, 2020	no	Cross-sectional	Patients with breast cancer	658	100	88.9		9	ISI	PHQ GAD
198	Yu [215]	2020	China	6 - 20 April 2020	yes	Cross-sectional	General population	1138	65.6	49.1		9	ISI	0
201	Wang [216]	2020	China	February 4 to February 18, 2020	no	Cross-sectional	General population	6437	56.13	38.99		9	PSQI	0
213	Blekas [217]	2020	Greek	April 10 until April 13, 2020.	no	Cross-sectional	Healthcare workers	270	73.7		18 to above 75	9	AIS	PHQ
218	Khanal [218]	2020	Nepal	April 26 and May 12, 2020	no	Cross-sectional	Healthcare workers	475	52.6	37.1	28.2	8	ISI	HADS
231	Liang [219]	2020	China	14 February to 29 March 2020	no	Cross-sectional	General population + healthcare professionals	2003	74.79	52.32	18 to above 60	8	ISI	PHQ GAD
232	Wankowicz [220]	2020	Poland	3 to 17 May 2020.	no	Cross-sectional	Healthcare workers	441	52.15		40	9	ISI	PHQ GAD
240	Pieh [221]	2020	Austria	10th of April 2020 for 10 days	yes	Cross-sectional	General population	733	49.9	55	18 to above 65	9	ISI	PHQ GAD
272	Alessi [222]	2020	Brazil	0	no	Cross-sectional	Patients with type 1 and type 2 diabetes	120	55.8		54.8	9	Mini Sleep Questionnaire (MSQ),	0
274	Huang [223]	2020	China	February 14 to March 29, 2020	no	Cross-sectional	General population	1172	69.28	39.51	18-40	9	ISI	0
275	McCracken [224]	2020	Sweden	May 14 and June 11, 2020	no	Cross-sectional	General population	1212	73.8	55.9	18 to 88	8	ISI	PHQ GAD
277	Parlapani [225]	2020	Greece	0	no	Cross-sectional	General population	103	61.17		69.85	9	AIS	PHQ GAD
278	Barrea [226]	2020	Italy	January 2020 to 30 April 2020	yes	Cross-sectional	General population	121	65.5		44.9	9	PSQI	0
283	Wańkowicz [227]	2020	Poland	3-17 May 2020	no	Cross-sectional	People with/ without Systemic Lupus Erythematosus	723	67.75		39.05	9	ISI	PHQ GAD
292	Dai [228]	2020	China	February 23-26, 2020	no	Cross-sectional	COVID-19 patients	307	43.32	81.76		9	PSQI	SDS SAA
239	Lin [57]	2020	Iran	February 15-30 2020	no	Cross-sectional	General population	1078	58.3		26.24	9	ISI	HADS
375	Ahorsu [229]	2020	Iran	1- 30 April 2020	no	Cross-sectional	General population	413	38	87.9	57.72	9	ISI	PHQ

### 3.3 Quality assessment

**Figure 2:**
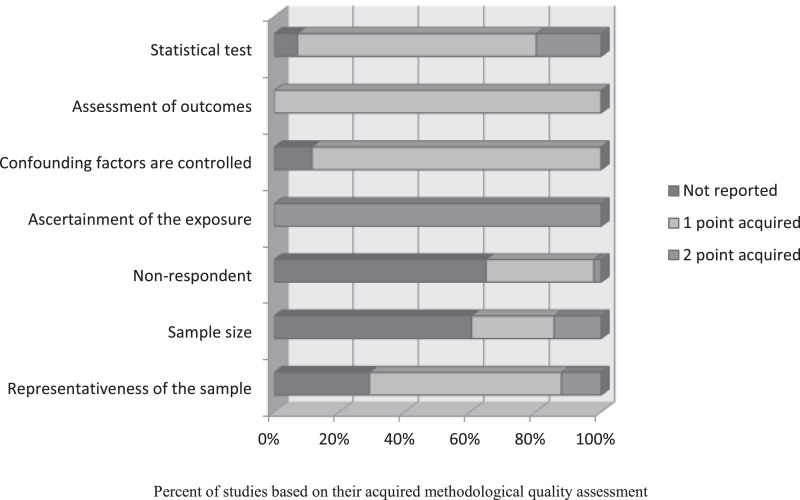
Results of quality assessment

As aforementioned, the maximum score on the NOS is 9 and a score less than 5 is classified as having a high risk of bias [46]. Based on this criterion, 130 studies were categorized as being high quality studies. The impacts of study quality were further assessed and reported in subgroup analysis. The most common problems were in selection of participants. Online sampling leads to non-representativeness of the sample, sample size being not estimated or justified, and number of non-respondents being not reported. The results of the quality assessment are provided in Figure 2.

### 3.4 Outcome measures

Three target groups of participants were studied: healthcare professionals (n=62), general population (n= 105), and COVID-19 patients (n=10). Outcome measures are reported based on these target groups.

#### 3.4.1 Sleep problems pooled prevalence based on participant target groups

##### 3.4.1.1 Healthcare professionals

**Figure 3:**
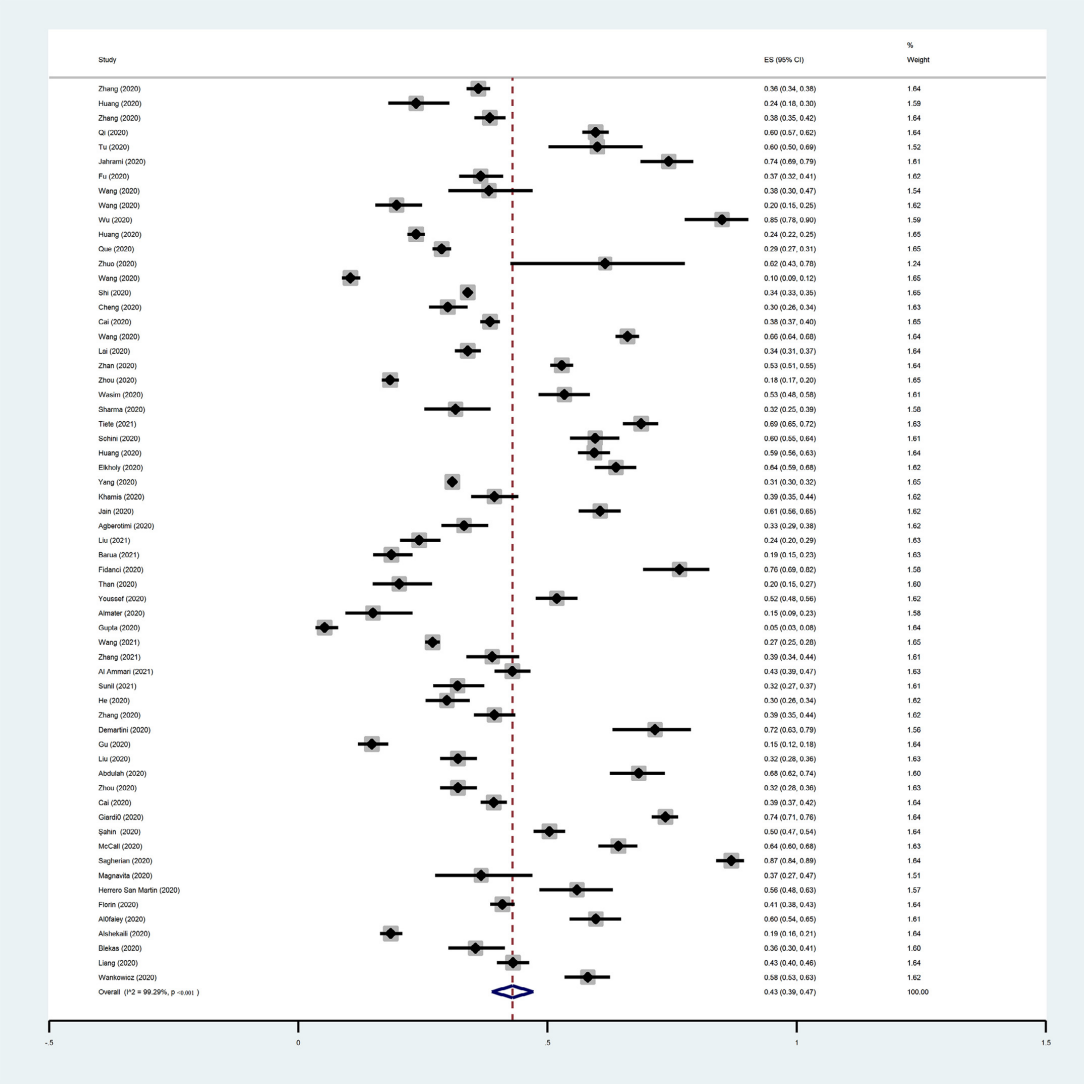
Forest plot displaying the estimated pooled prevalence of sleep problems among health professionals

**Figure 4:**
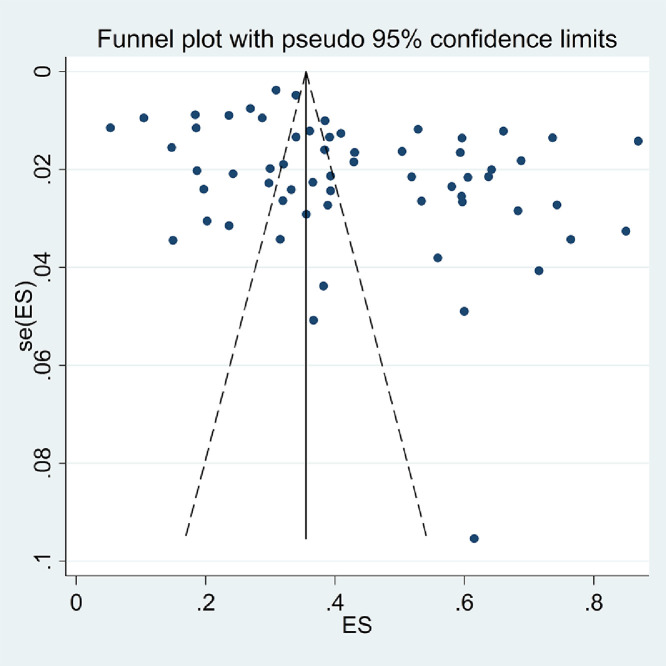
Funnel plot assessing publication bias in studies regarding prevalence of sleep problems among health professionals

**Figure 5:**
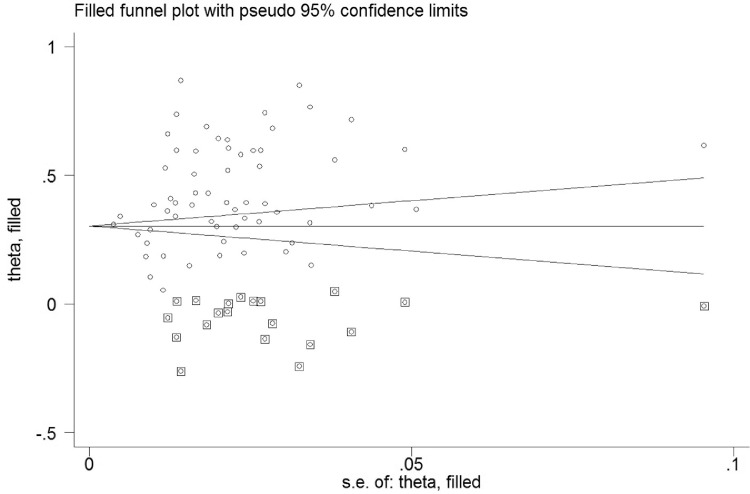
Corrected funnel plot assessing publication bias in studies regarding prevalence of sleep problems among health professionals

The pooled estimated prevalence of sleep problems among healthcare professionals was 43% [95% CI: 39-47%, I^2^:99.29%, Tau^2^:0.03]. Figure 3 provides the forest plot showing the pooled prevalence. Subgroup analysis (Table 2) and uni-variable meta-regression (Table 3), and multivariable meta-regression (Table 4) showed that none of the examined variables influenced the prevalence of sleep problems or heterogeneity. The probability of publication bias was assessed using Begg's test and funnel plot. Based on Begg's test (*p*=0.12) and funnel plot (Figure 4), the probability of publication bias was confirmed. Due to probability of publication bias in estimation of pooled prevalence of sleep problems in healthcare professions, the fill-and-trim method was used to correct the results. In this method, 20 studies were imputed and the corrected results based on this method showed that pooled prevalence of sleep problems among healthcare professions was 0.31 (95% CI: 0.27 to 0.36; *p*<.001). Funnel plot after trimming is provided in Figure 5. Also, sensitivity analysis showed that pooled effect size was not affected by a single study effect.

**Table 2:** Results of subgroup analysis regarding estimated pooled prevalence

		*Healthcare professionals (N=62)*				*General Population (N=105)*				*Covid-19 patients (N=10)*			
Variable		No. of studies	Pooled prevalence (95% CI)	I^2^ (%)	*p* for I^2^	No. of studies	Pooled prevalence and 95% CI	I^2^ (%)	*p* for I^2^	No. of studies	Pooled prevalence and 95% CI	I^2^ (%)	*p* for I^2^
Quality	Low quality	17	41 (33-48)	98.99	0.47	23	33 (27-39)	99.61	0.10	3	42 (27-57)	97.8	0.04
	High quality	45	44 (39-49)	99.37		82	38 (35-42)	99.76		7	64 (49-71)	-	
Lockdown period	Yes	3	45 (32-57)	-	0.81	29	46 (37- 55)	99.79	0.01	-	-	-	-
	No	59	43 (39-47)	99.32		76	34 (31-37)	99.71		10	57 (42- 72)	98.5	
Gender group	Female only	21	40 (34-47)	99.33	0.34	32	34 (30-38)	99.74	0.11	1	82 (78- 85)	-	<0.001
	Both gender	41	44(39-50)	99.28		73	39 (35- 43)	99.75		9	54 (40 -69)	98.10	
Study design	Cross Sectional	60	42 (38-47)	99.3	0.96	99	36 (33-39)	99.77	<0.001	9	57 (41-73)	98.67	0.80
	Case-control	2	42 (41-44)	-		2	50 (32-38)	-		-	-	-	
	Longitudinal	-	-	-		4	63 (52-74)	86.86		1	55 (47-63)	-	
Measure of sleep	PSQI	19	48 (38-58)	99.29	0.24	38	45 (39-50)	99.73	<0.001	3	65 (42- 88)	-	<0.001
	ISI	34	39 (34-45)	99.37		53	31 (28-35)	99.75		6	48 (38- 58)	92.81	
	other	9	46 (35-56)	98.12		14	39 (29-49)	99.68		1	82 (78-85)	-	
Overall estimated prevalence		62	43 (39-47)	99.29		105	37 (35-40)	99.75		10	57 (42- 72)	98.5	

95% CI=95% confidence interval. PSQI=Pittsburgh Sleep Quality Index. ISI=Insomnia Severity Index.

**Table 3:** Results of Univariable meta-regression regarding estimated pooled prevalence

	*Healthcare professionals (N=62)*							*General Population (N=105)*							*Covid-19 patients (N=10)*						
Variable	No. of studies	Coeff.	S.E.	*p*	I^2^ res. (%)	Adj. R^2^ (%)	Tau^2^	No. of studies	Coeff.	S.E.	*p*	I^2^ res. (%)	Adj. R^2^ (%)	Tau^2^	No. of studies	Coeff.	S.E.	*p*	I^2^ res. (%)	Adj. R^2^ (%)	Tau^2^
Country	62	0.002	0.002	0.38	99.26	-0.26	0.04	105	0.006	0.001	<0.001	99.68	12.34	0.04	10	-0.004	0.01	0.77	98.64	-11.13	0.04
Age	34	0.005	0.007	0.46	99.2	-1.5	0.04	69	0.002	0.002	0.48	99.8	-0.7	0.04	8	0.0005	0.003	0.88	98.66	-12.57	0.04
Female % of participants	62	0.001	0.001	0.72	99.29	-1.45	0.04	103	-0.0001	0.001	0.95	99.73	-0.9	0.04	10	-0.002	0.006	0.71	98.65	-10.51	0.04
Married % of participants	39	0.001	0.002	0.51	99.30	-1.54	0.04	52	0.001	0.001	0.37	99.74	-0.4	0.04	8	-0.002	0.007	0.80	98.46	-16.04	0.04

Coeff.=coefficient. S.E.=standard error. I^2^ res.=I^2^ residual. Adj. R^2^=adjusted R^2^.

**Table 4:** Results of multivariable meta-regression regarding estimated pooled prevalence

	*Healthcare professionals*			*General Population*		
Variable	Coefficient	S.E.	*p*	Coefficient	S.E.	*p*
Country	-0.003	0.007	0.64	0.006	0.001	<0.001
Design	0.06	0.24	0.81	⁎⁎		
Lockdown period (yes vs. no)	0.23	0.17	0.21	0.08	0.04	0.03
Study quality (low vs. high quality)	0.12	0.13	0.40	0.04	0.04	0.39
Age	-0.003	0.01	0.78	0.001	0.001	0.26
% Female of participants	0.03	0.003	0.39	0.001	0.001	0.30
% Married of participants	0.003	0.004	0.35	-0.001	0.001	0.11
Measure of sleep	-0.06	0.09	0.50	-0.03	0.032	0.20
Between-study variance (tau^2^)	0.03			0.03		
% Residual variation due to heterogeneity (I^2^ residual)	99.27			99.68		
Proportion of between-study variance explained (adjusted R^2^)	-26.23			26.33		

N.B. Due to insufficient observations, meta-regression was not conducted for COVID-19 patients subgroup.

⁎⁎ Due to collinearity design was omitted.

##### 3.4.1.2 General population

**Figure 6:**
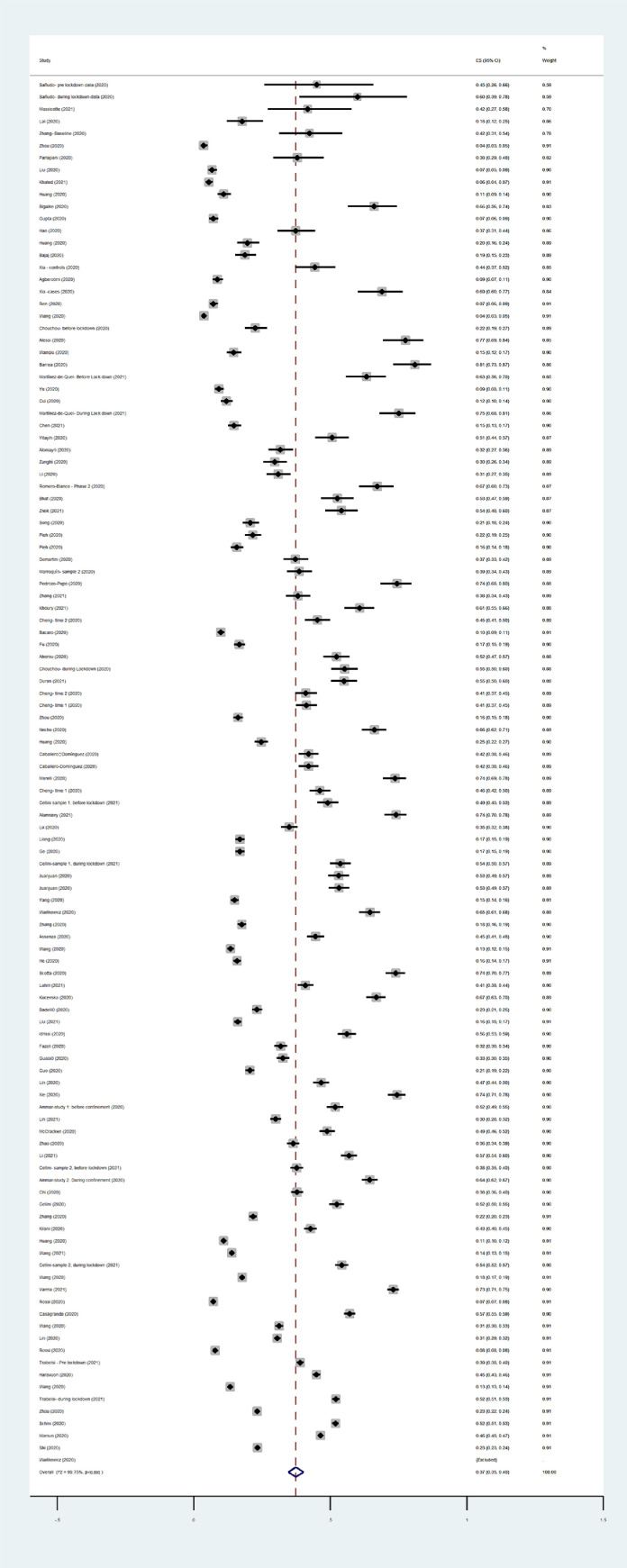
Forest plot displaying the estimated pooled prevalence of sleep problems among general population

The pooled estimated prevalence of sleep problems among the general population was 37% [95% CI: 35-40%, I2:99.77%, Tau^2^:0.02]. Figure 6 provides the forest plot showing the pooled prevalence. Subgroup analysis (Table 2) showed that during lockdown, participants in longitudinal studies showed a significantly higher prevalence of sleep problems. Based on uni-variable meta-regression (Table 3), the country of residence was the other significant variable in prediction of prevalence of sleep problems among the general population. Also, multivariable meta-regression (Table 4) confirmed that country and lockdown period were significant influential factors on prevalence of sleep problems, explaining 26.32% of variance.

**Figure 7:**
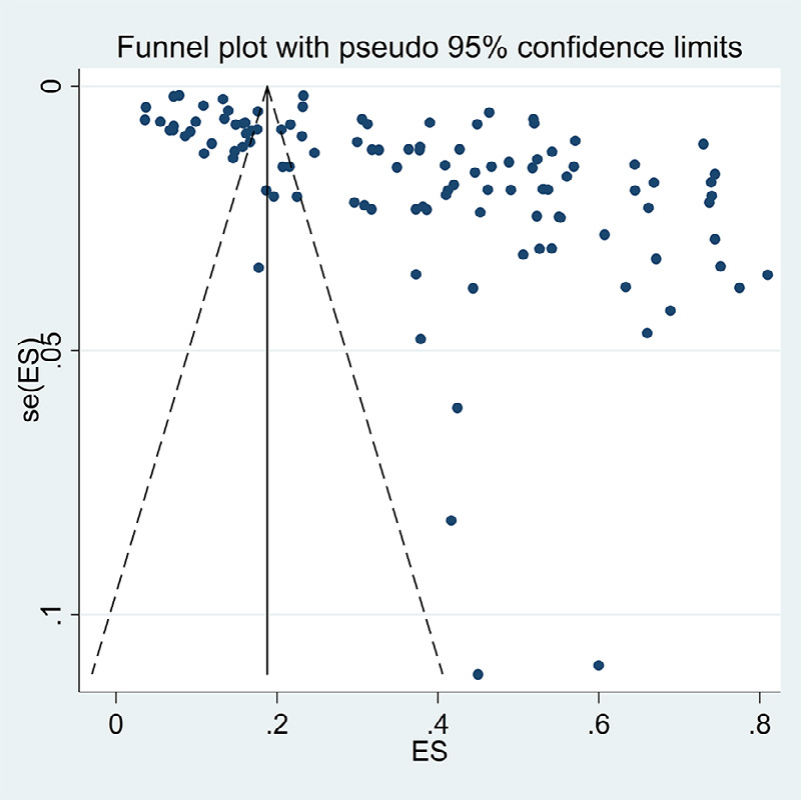
Funnel plot assessing publication bias in studies regarding prevalence of sleep problems among general population

**Figure 8:**
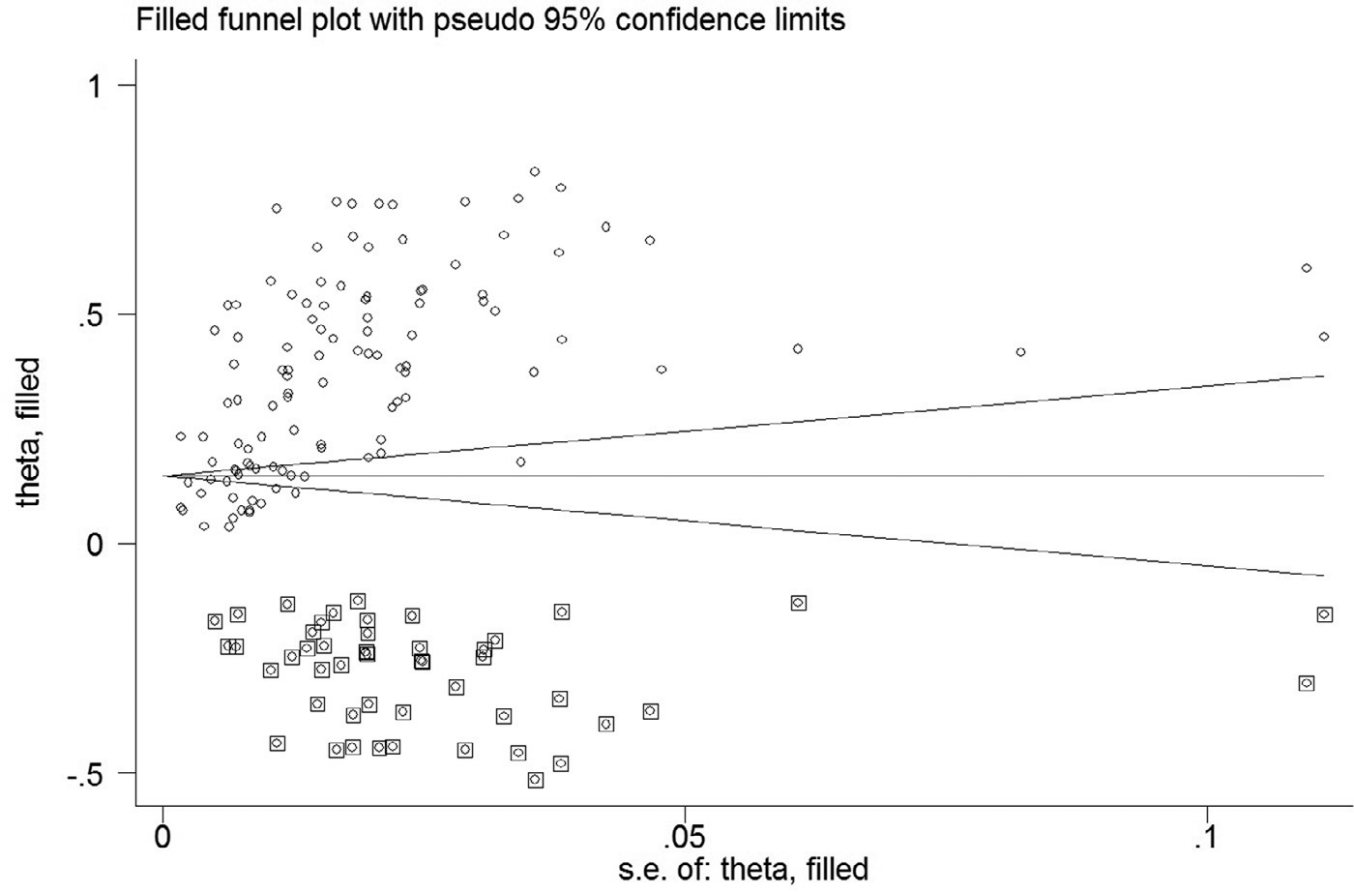
Corrected funnel plot assessing publication bias in studies regarding prevalence of sleep problems among general population

The probability of publication bias was assessed using Begg's test and funnel plot. Based on Begg's test (*p*=0.01) and funnel plot (Figure 7), the probability of publication bias was confirmed. Due to probability of publication bias in estimation of pooled prevalence of sleep problems among the general population, the fill-and-trim method was used to correct the results. In this method, 50 studies were imputed and the corrected results based on this method showed that pooled prevalence of sleep problems was 18% (95% CI: 15-21%; *p*<.001). Funnel plot after trimming is provided in Figure 8. Also, sensitivity analysis showed that pooled effect size was not affected by a single study effect.

##### 3.4.1.3 COVID-19 patients

**Figure 9:**
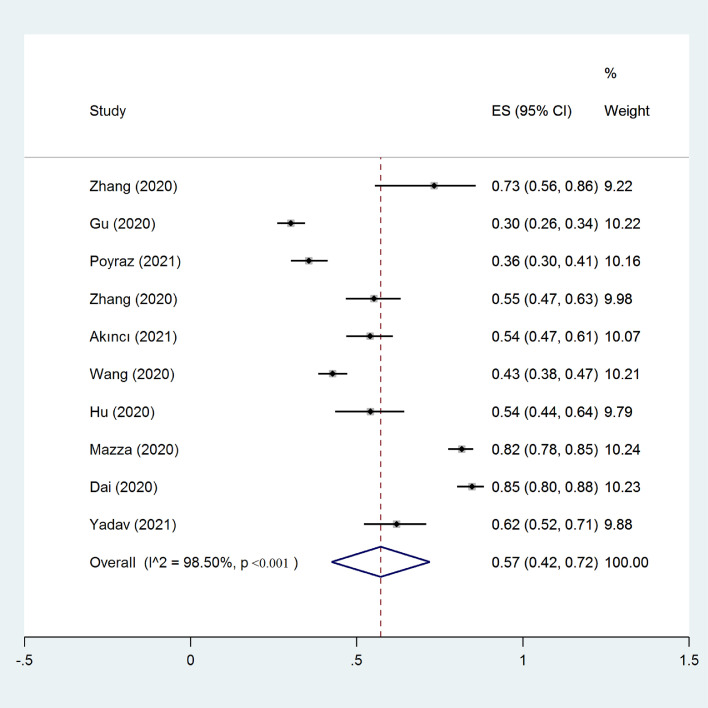
Forest plot displaying the estimated pooled prevalence of sleep problems among COVID-19 patients

**Figure 10:**
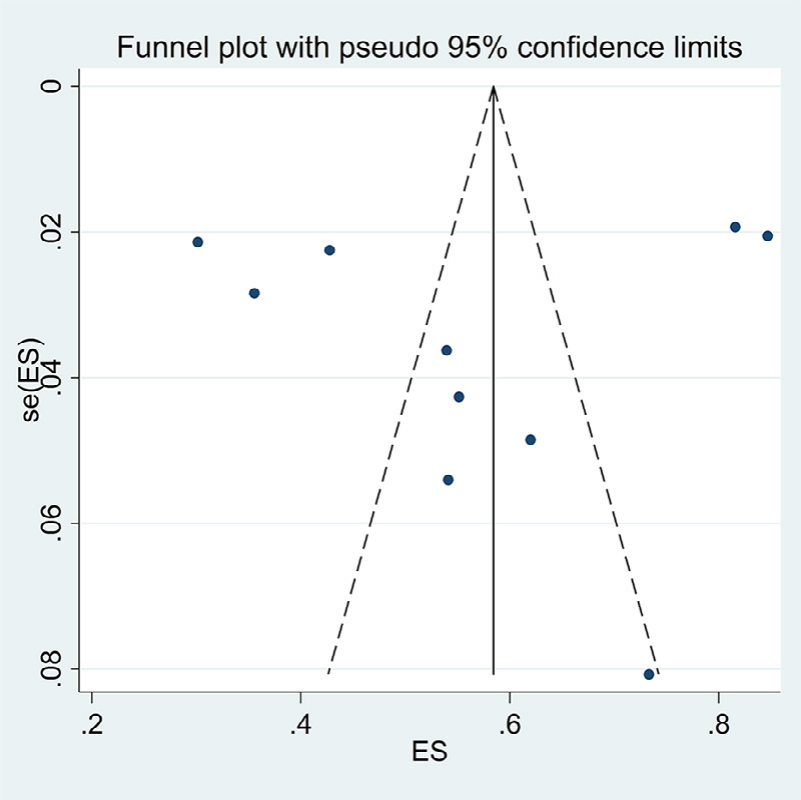
Funnel plot assessing publication bias in studies regarding prevalence of sleep problems among Covid patients

The pooled estimated prevalence of sleep problems was 57% among COVID-19 patients [95% CI: 42 to 72%, I^2^:98.5%, Tau^2^:0.06]. Figure 9 provides the forest plot showing the pooled prevalence. Subgroup analysis (Table 2) showed studies with female-only participants had a higher prevalence of sleep problems significantly (82% vs. 54% respectively). Other variables did not influence heterogeneity or estimated pooled prevalence in this participants group. The probability of publication bias was assessed using Begg's test and funnel plot. Based on Begg's test (*p*=0.53) and funnel plot (Figure 10), the probability of publication bias was rejected. Also, sensitivity analysis showed that pooled effect size was not affected by a single study effect.

Overall, the prevalence of sleep problems was significantly different in target participants considering 95% confidence interval of sleep prevalence. The corrected pooled estimated prevalence of sleep problems was 31% (95% CI: 27-36%), 18% (95% CI: 15-21%) and 57% (95% CI: 42-72%), among healthcare professional, general population and COVID-19 patients respectively. The highest prevalence of sleep problems was seen among COVID-19 patients.

#### 3.4.2 Association of sleep problems with psychological distress

##### 3.4.2.1 Healthcare professionals

**Figure 11:**
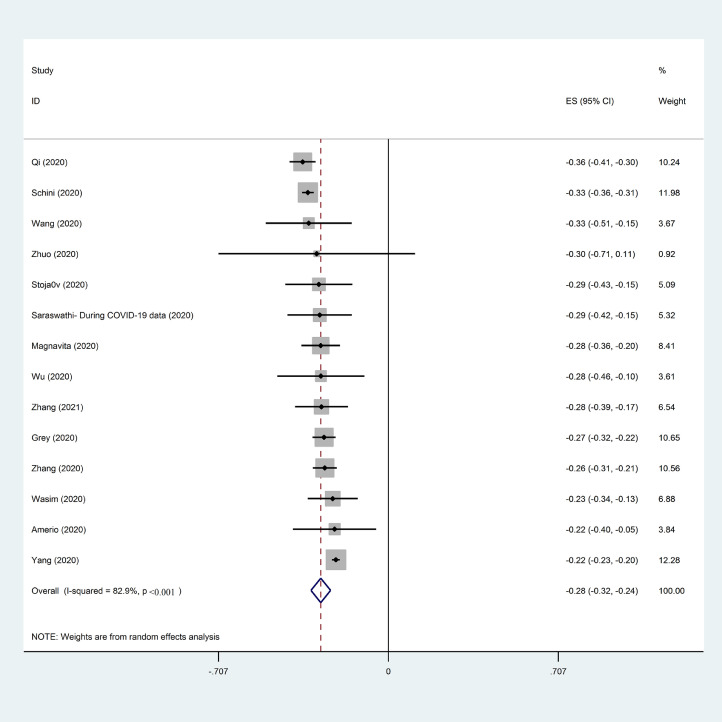
Forest plot displaying the estimated pooled Fishers’ Z score in association of sleep problems and depression among health professionals

**Figure 12:**
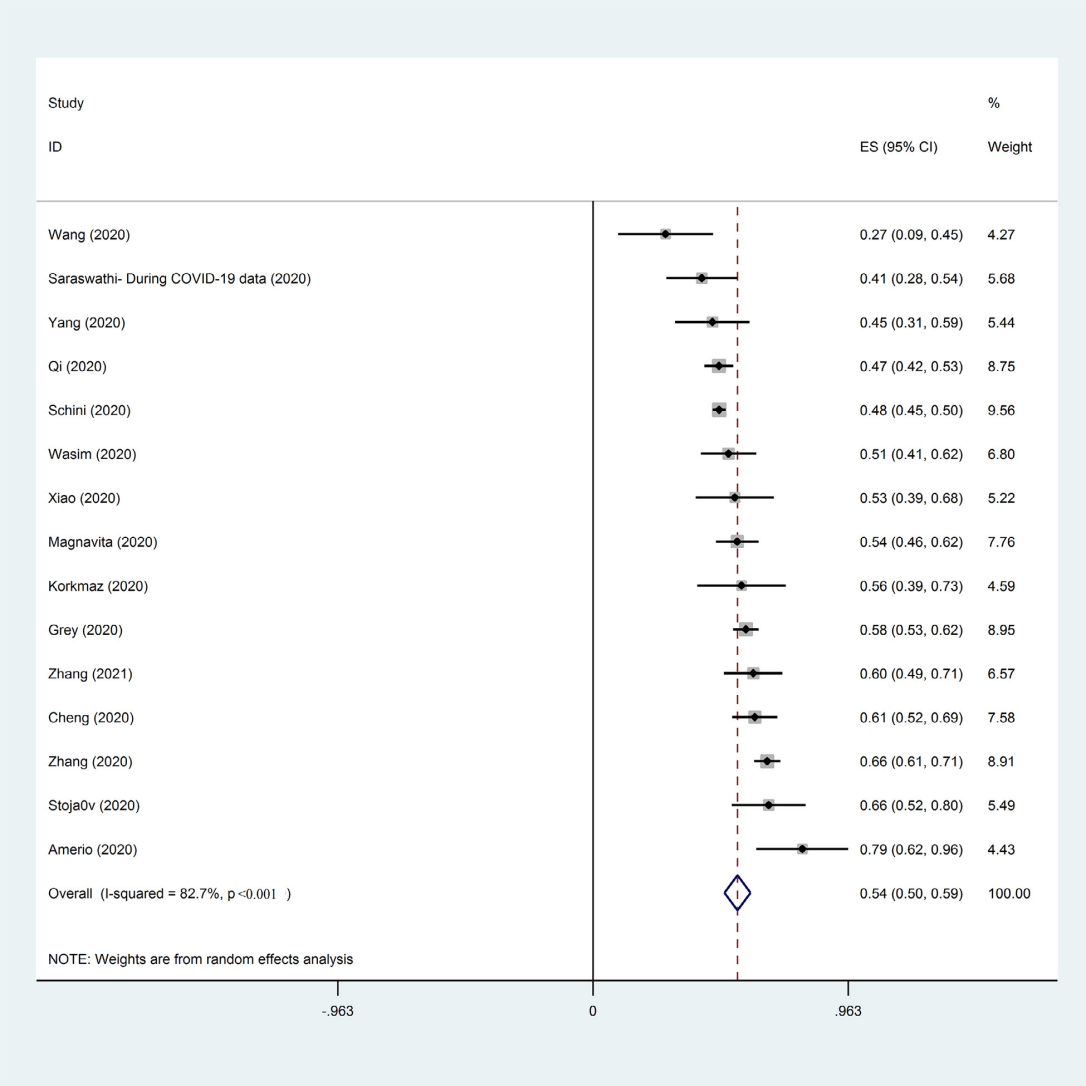
Forest plot displaying the estimated pooled fishers’ Z score in association of sleep problems and anxiety among health professionals

The association of sleep problems with depression and anxiety among health professionals were reported in 14 and 15 studies respectively. The pooled estimated effect size showed poor correlation between sleep problems and depression with Fisher's z score of -0.28 [95% CI: -0.32 to -0.24, *p*<0.001, I^2^=82.9%; Tau^2^ = 0.004]. However, a moderate correlation was found between sleep problems and anxiety with Fisher's z score of 0.55 [95% CI: 0.49 to 0.59, *p*<0.001, I^2^=82.7%; Tau^2^ = 0.10]. The forest plots are shown in Figure 11, Figure 12.

Based on subgroup analysis (Table 5), quality of studies (low vs. high), gender group of participants (female vs. both gender), and measure of sleep problems (PSQI vs. others) influenced heterogeneity of association of sleep problems and depression among health professionals. Meta-regression (Table 7) showed that age and marital status (married vs. others) significantly decreased the heterogeneity and explained substantial proportion of variance (72.8% and 43.85% respectively). Examined variables in subgroup analysis and meta-regression were not identified as possible source of heterogeneity or influential in the estimated pooled effect size in the association of sleep problems and anxiety (Table 6). Publication bias and small study effect was not found in association of sleep problems and depression/anxiety based on Begg's test (*p*=0.87 and *p*=0.81 respectively).

**Table 5:** Results of subgroup analysis regarding estimated pooled correlation between sleep and **Depression**

		*Healthcare professionals (N=14)*			*General Population(N=15)*		
Variable		No. of studies	ES (95% CI)	I^2^ (%)	No. of studies	ES (95% CI)	I^2^ (%)
Quality	Low quality	6	-0.30 (-0.35; -0.25)	28	4	-0.32 (-0.37; -0.26)	71.2
	High quality	8	-0.28 (-0.33; -0.22)	88.9	11	-0.29 (-0.32; -.27)	76.2
Gender group	Female only	6	-0.30(-0.34; -0.26)	23.8	4	-0.32 (-0.39; -0.25)	79.7
	Both gender	8	-0.27 (-0.32; -0.21)	88.7	11	-0.29 (-0.32; -0.27)	74.7
Lockdown	Yes	1	-0.34 (-0.36; -0.31)	-	4	-0.33 (-0.38; -0.28)	78.6
	No	13	-0.27 (-0.31; -0.24)	60.8	11	-0.29 (-0.31; -0.26)	58.9
Study design	Cross-sectional	12	-0.28 (-0.32; -0.24)	85.5	14	-0.30 (-0.32; -0.27)	75.5
	Case-control	1	-0.28 (-0.46; -0.1)	-	-	-	-
	Longitudinal	1	-0.29 (-0.42; -0.15)	-	1	-0.38 (-0.51; -0.24)	-
Measure of sleep	PSQI	7	-0.30 (-0.34; -0.27)	4.6	7	-0.30 (-0.33; -0.27)	64.6
	ISI	5	-0.22 (-0.24; -0.21)	-	7	-0.29 (-0.33; -0.25)	72.9
	other	2	-0.32 (-0.37; -0.28)	35	1	-0.34 (-0.36; -0.31)	-
Overall estimated prevalence		14	-0.28 (-0.32; -0.24)	82.9	15	-0.30 (-0.32; -0.28)	74.4

**Table 6:** Results of subgroup analysis regarding estimated pooled correlation between sleep and **Anxiety**

		*Healthcare professionals (N=15)*			*General Population(N=12)*		
Variable		No. of studies	ES (95% CI)	I^2^ (%)	No. of studies	ES (95% CI)	I^2^ (%)
Quality	Low quality	7	0.59 (0.49; 0.68)	82.5	3	0.55 (0.48; 0.62)	73.5
	High quality	8	0.52 (0.46; 0.58)	78.1	9	0.53 (0.46; 0.61)	96.2
Lockdown period	Yes	1	0.48 (0.45; 0.50)	-	3	0.45 (0.32; 0.58)	78.4
	No	14	0.55 (0.50; 0.60)	75.6	9	0.57 (0.49; 0.65)	96.3
Gender group	Female only	7	0.55 (0.47; 0.63)	83.9	3	0.49 (0.31; 0.66)	90.9
	Both gender	8	0.54 (0.48; 0.60)	76.8	9	0.56 (0.47; 0.64)	95.8
Study design	Cross-sectional	14	0.55 (0.50; 0.60)	83.3	11	0.56 (0.49; 0.62)	95.4
	Case-control	-	-	-	-	-	-
	Longitudinal	1	0.41 (0.28; 0.55)	-	1	0.28 (0.15; 0.42)	-
Measure of sleep	PSQI	10	0.53 (0.47; 0.58)	68.1	6	0.51 (0.47; 0.57)	88.7
	ISI	2	0.64 (0.51; 0.77)	60.1	5	0.60 (0.40; 0.80)	97.7
	Other	3	0.50 (0.44; 0.56)	78.1	1	0.48 (0.45; 0.50)	-
Overall estimated prevalence		15	0.55 (0.49 to 0.59)	82.7	12	0.54 (0.48; 0.60)	95.2

##### 3.4.2.2 General population

**Figure 13:**
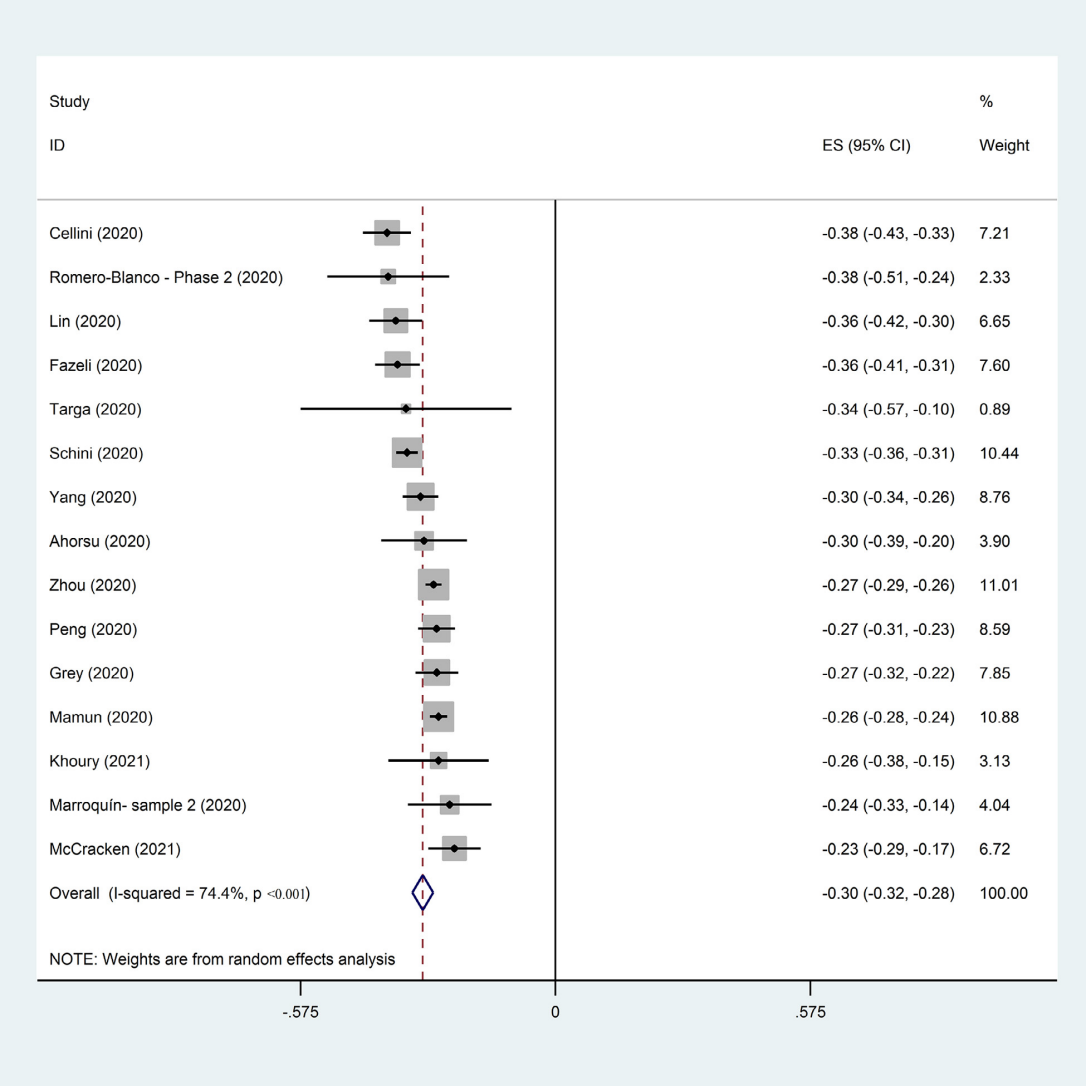
Forest plot displaying the estimated pooled Fishers’ Z score in association of sleep problems and depression among general population

**Figure 14:**
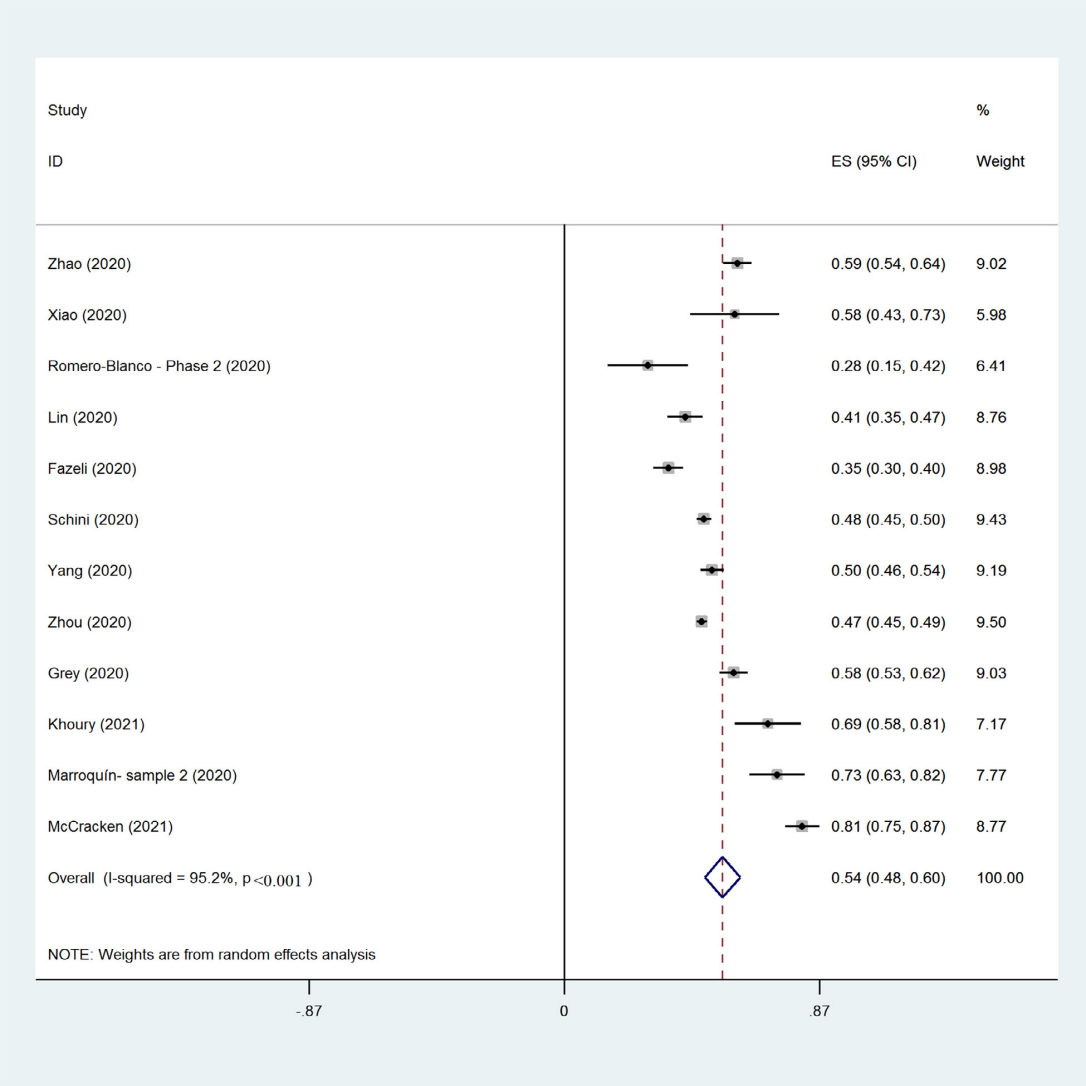
Forest plot displaying the estimated pooled Fishers’ Z score in association of sleep problems and anxiety among general population

The association of sleep problems with depression and anxiety among the general population were reported in 15 and 12 studies respectively. The pooled estimated effect size showed moderate correlation between sleep problems and depression with Fisher's z score of -0.30 [95% CI: -0.32 to -0.28, *p*<0.001, I^2^=74.4%; Tau^2^ = 0.001]. Also, a moderate correlation was found between sleep problems and anxiety with Fisher's z score of 0.54 [95% CI: 0.48 to 0.60, *p*<0.001, I^2^=95.2%; Tau^2^ = 0.01]. The forest plots are shown in Figure 13, Figure 14. Based on subgroup analysis (Table 5 and 6), lockdown status (no vs. yes) reduced the heterogeneity in association of sleep problems and depression. Based on meta-regression (Table 7), age was a significant moderator in association between sleep problems and anxiety, which explained 50.37% of variance. However, the other examined variables were not identified as possible sources of heterogeneity or influential on the estimated pooled effect size in the association between sleep problems and depression/anxiety.

**Table 7:** Results of meta-regression regarding correlation between sleep and psychological distress

Depression	Hea*lthcare professionals (N=14)*							*General Population(N=15)*						
Variable	No of studies	Coeff.	S.E.	*p*	I^2^ res. (%)	Adj. R^2^ (%)	Tau^2^	No of studies	Coeff.	S.E.	*p*	I^2^ res. (%)	Adj. R^2^ (%)	Tau^2^
Country	14	0.002	0.003	0.62	83.99	-8.4	0.002	15	-0.0004	0.001	0.64	75.9	-7.49	0.002
Age	12	-0.002	0.001	0.006	13.91	72.8	0.0004	13	0.002	0.001	0.21	77.65	1.88	0.002
Female % of participants	14	-0.002	0.001	0.12	71.23	19.31	0.001	15	-0.001	0.001	0.38	68.46	3.93	0.002
Married % of participants	12	-0.001	0.0004	0.08	37.2	43.85	0.001	6	-0.001	0.0004	0.52	72.16	-3.47	0.002

Anxiety	*Healthcare professionals (N=15)*							*General Population (N=12)*						
	No of studies	Coeff.	S.E.	*p*	I^2^ res. (%)	Adj. R^2^ (%)	Tau^2^	No of studies	Coeff.	S.E.	*p*	I^2^ res. (%)	Adj. R^2^ (%)	Tau^2^
Country	15	-0.002	0.005	0.73	83.61	- 13.03	0.01	12	-0.0005	0.003	0.89	95.62	-10.62	0.02
Age	10	0.011	0.005	0.05	62.52	54.77	0.01	10	0.01	0.005	0.02	95.03	50.37	0.02
Female % of participants	15	-0.002	0.002	0.38	83.37	- 12.64	0.01	11	0.001	0.003	0.70	95.68	-9.10	0.03
Married % of participants	9	0.006	0.003	0.46	87.86	21.25	0.01	5	0.0004	0.005	0.95	97.31	-31.77	0.02

Based on Begg's test, publication bias and small study effect were not found in the association between sleep problems and depression (*p*=0.52). Although publication bias was not significant in association between sleep problems and anxiety (*p*=0.41), based on funnel plot, publication bias was probable. Consequently, fill and trim method was used to correct probable publication bias. After imputation of three studies, the association between sleep problems and anxiety was estimated as Fisher's z score of 0.48 (95% CI: 0.41 to 0.54).

#### 3.4.3 COVID-19 patients

**Figure 15:**
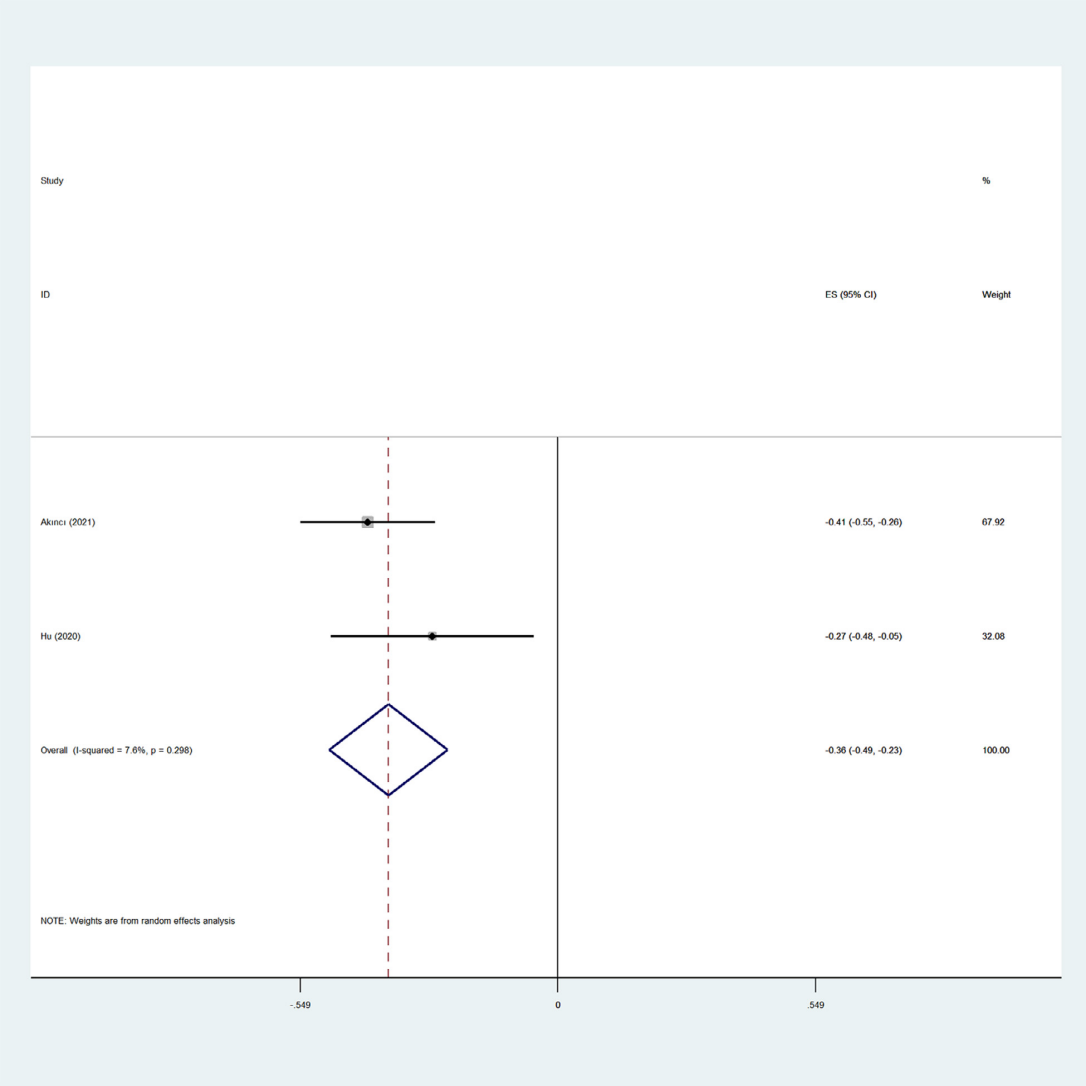
Forest plot displaying the estimated pooled Fishers’ Z score in association of sleep problems and depression among COVID-19 patients

**Figure 16:**
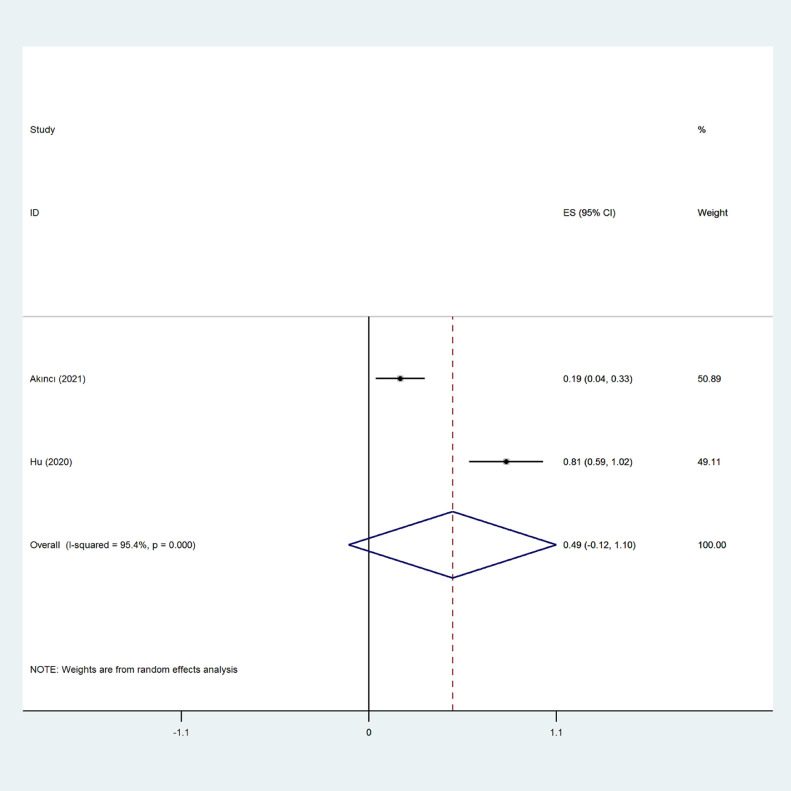
Forest plot displaying the estimated pooled fishers’ Z score in association of sleep problems and anxiety among Covid patients

The association of sleep problems with depression and anxiety among general population was reported in only two studies. The pooled estimated effect size showed moderate correlation between sleep problems and depression with Fisher's z score of -0.36 [95% CI: -0.49 to -0.24, *p*=0.0007, I^2^=7.4%; Tau^2^ = 0.001]. Also, a moderate correlation was found between sleep problems and anxiety with Fisher's z score 0.49 [95% CI: -0.12 to 1.1, *p*<0.001, I^2^=95.2%; Tau^2^ = 0.01]. The forest plots are shown in Figure 15, Figure 16. The number of studies was too few to conduct further secondary analysis including subgroup/meta-regression analysis, controlling publication bias, and small study effect.

## 4 Discussion

The present systematic review and meta-analysis synthesized data from 177 recently published studies on this topic to more rigorously investigate the prevalence of sleep problems and how sleep associated with psychological distress. The synthesized results showed that the pooled estimated prevalence of sleep problems regardless of gender and population was 37% during the COVID-19 outbreak. Additionally, a much higher prevalence rate of sleep problems was identified among patients with COVID-19 infection (55%) and healthcare professionals (43%). These findings concur with Jahrami et al. [38] who reported in their meta-analysis that the highest prevalence rate of sleep problems was found among COVID-19 patients. Meta-regression in the present review further indicated that country, age, gender, and marital status did not contribute to the estimated prevalence in sleep problems.

The nonsignificant finding for gender contradicts prior evidence showing that being female is a risk factor for insomnia and mental health problems [27, 56]. This may be explained by the samples recruited because the analyzed studies in the present review comprised a large proportion of females. The imbalanced gender distribution may have led to a reduced gender effect, which in turn, resulted in a nonsignificant finding. Regarding the association between sleep problems and psychological distress, sleep problems were found to be moderately correlated with depression (ES=0.54) and anxiety (ES=0.55). Subgroup analysis and meta-regression additionally showed that being a COVID-19 patient and being of older age were significant predictors of a higher association between sleep problems and psychological distress.

The high prevalence of sleep problems found in the present review can be explained by fear of COVID-19 and sleep-related factors (e.g., the changes in sleep-wake habits with delayed bedtime, lights off time, and sleep onset time due to quarantine and lockdown) [57]. The national and global COVID-19 death statistics are commonly and routinely reported by the social media and news [57]. Therefore, prior research has found the higher levels of psychological distress and significant symptoms of mental illness in various populations since the start of the pandemic [4], [5], [6]. Indeed, evidence prior to the pandemic has demonstrated that individuals may experience sleep problems when they experience major public health threats [16], [17], [18]. The higher prevalence of sleep problems found among healthcare professionals can be further explained by their job nature. Health professionals, especially those who are frontline workers dealing with COVID-19 infected patients on a daily basis, encounter much higher high risk of infection and irregular work schedules than those working in other occupations [10], [11], [12], [13], [14], [15].

Lockdown was found to be a significant factor in explaining sleep problems. However, this finding may be confounded by the different policies implemented to inhibit the spread of COVID-19 across the 39 countries analyzed in the present review. For example, mainland China launched a strict lockdown policy to prohibit almost all outdoor activities, while the lockdown policy in other countries was not as strict. Nevertheless, the present findings support prior evidence that lockdown negatively impacted individuals’ psychological health and sleep [57].

There are several clinical implications from the present study's findings. First, government and healthcare providers worldwide need to design and implement appropriate programs and treatments to assist different populations, including healthcare professionals, patients, and the general population, in overcoming sleep problems. For example, effective programs (e.g., cognitive behavioral therapy for insomnia and meditation) [58] reported in prior research can be embedded in smartphone apps and healthcare professional training to prevent or deal with the sleep problems for different populations. Second, the associations between sleep problems and psychological distress provide the empirical evidence that healthcare providers should simultaneously tackle sleep problems and psychological distress. Consequently, psychological distress can be reduced when an individual's sleep is improved (and *vice versa*). Third, special attention may need to be paid to COVID-19 patients and older individuals because the present review showed a higher association between their sleep problems and psychological distress. Moreover, specific populations such as children and their caregivers should not be ignored regarding their psychological needs and sleep issues. Although the present review did not provide evidence on pediatric populations, the present findings concerning the specific group of older individuals may generalize to other specific populations. It is recommended that programs comprising psychological support for family having children to overcome the difficulties during COVID-19 pandemic are implemented [60].

The present review has some strengths. First, the prevalence of sleep problems has been estimated across different populations and this information provides healthcare providers with a greater and more contextualized picture regarding the impacts of COVID-19 on sleep problems. Second, methodological quality of each analyzed study was assessed using the NOS checklist. Within the meta-analysis findings, subgroup analysis and meta-regression were used to provide thorough information and therefore the meta-analysis findings are robust. Third, generalizability of the present review's findings is good because the synthesized sample size was large (N=345,270) and the participants were recruited from 39 countries.

The present review has some limitations. First, most of the studies adopted a cross-sectional design (n=56) and only seven studies (three which used a case-control design and four which used a longitudinal design) considered the *time* effect in the causal relationship. Therefore, the relationships between sleep problems and psychological distress found in the present review do not have strong causality evidence. Diverse evidence in the causality has been proposed. Using longitudinal designs, Vaghela and Sutin [59] found that psychological distress might lead to poor sleep, while Mazzer and Linton [60] found that shorter sleep duration might lead to higher levels of psychological distress. Moreover, the lack of pre-COVID-19 pandemic information on sleep problems hinders the understanding of *changes* of sleep problems caused by COVID-19. Second, different measures were used in the studies that were evaluated (e.g., PSQI, ISI, and ASI for sleep problems). Given that different measures may have different features in capturing the severity of sleep problems, there may have some biases in estimating prevalence for sleep problems and effect sizes for the associations between sleep problem and psychological distress. All the studies evaluated here used self-report methods in assessing sleep problems and psychological distress. Therefore, findings in the present review cannot rule out social desirability and memory recall biases. Third, the impacts of COVID-19 on sleep and mental health problems are dynamic. That is, individuals may have different levels of sleep and mental health problems according to the severity of COVID-19 outbreak in their localities or countries. Moreover, the policies in controlling the COVID-19 outbreak are different across countries [57,[61], [62], [63], [64], [65], [66]]. Therefore, the estimated findings in the present review cannot represent the impacts of COVID-19 during a specific period. Fourth, the analyzed studies in the present review had a large proportion of Chinese and Italian populations. Similarly, the synthesized samples were mostly young adults. Therefore, the generalizability of the present review's findings to different ethnic populations and age groups (i.e., older people and children) is restricted. Given that China and Italy were the first two countries to be severely impacted by the COVID-19 pandemic, there is understandably more research carried out in these two countries. However, the contributions of other countries, especially the American and African populations, should not be ignored. Further research should be carried out in other ethnic populations and different countries to balance the findings and maximize the generalizability. Fifth, the present meta-analysis had very large heterogeneity (as shown in Fig. 3) and evidence of publication bias (as shown in Fig. 4). Therefore, the findings without removing the heterogeneity in the meta-regression and subgroup analysis might be biased. Finally, most of the studies included in the meta-analysis were not of high quality (as shown in Fig. 2). Therefore, future studies require higher quality designs to investigate sleep problems during COVID-19 pandemic.

In conclusion, sleep problems appear to have been common during the COVID-19 pandemic. One in every three individuals reported the sleep problems. Nearly half of the healthcare professionals (43%) encountered sleep problems during the pandemic period. Healthcare providers may want to design appropriate programs to help individuals overcome their sleep problems. Moreover, sleep problems were found to be associated with higher levels of psychological distress (including depression and anxiety). Therefore, with the use of effective programs treating sleep problems, psychological distress may be reduced. Vice versa, the use of effective programs treating psychological distress, sleep problems may be reduced. However, it is possible that the association between sleep problems and psychological distress found in the present review is contributed by confounders. In other words, causality may not be happened between sleep problems and psychological distress. Therefore, more longitudinal studies and randomized controlled trials are needed to investigate the causality between sleep problems and psychological distress.

## Declaration of Competing Interest

Chung-Ying Lin was supported in part by a research grant from the Ministry of Science and Technology, Taiwan (MOST109-2327-B-006-005). All other authors have nothing to declare.

## Appendix Supplementary materials

Image, application 1
