# Supplementary material for: Sleep problems during COVID-19 pandemic and its’ association to psychological distress: A systematic review and meta-analysis
Source: eClinicalMedicine. 2021 Jun 10;36:100916. doi: 10.1016/j.eclinm.2021.100916 (PMC8192091; doi:10.1016/j.eclinm.2021.100916)
Supplement: Supplementary file 1 [file mmc1.docx]

Supplementary Table 1: The full search strategy for each database.

| Database | Search Date | No of retrieved records | Syntax |
| --- | --- | --- | --- |
| PubMed central | 338 | February 17, 2021 | ("2019 nCoV" OR 2019nCoV OR "2019 novel coronavirus" OR "COVID 19" OR COVID19 OR "new coronavirus" OR "novel coronavirus" OR "SARS CoV-2" OR (Wuhan AND coronavirus) OR "COVID 19" OR "SARS-CoV" OR "2019-nCoV" OR "SARS-CoV-2") AND (("insomnia" OR "sleep problems" OR "Sleep quality" OR Dyssomnia OR ("Sleep Disorders" AND Extrinsic) OR "Extrinsic Sleep Disorder" OR "Extrinsic Sleep Disorders" OR ("Sleep Disorder" AND Extrinsic) OR "Limit-Setting Sleep Disorder" OR "Limit Setting Sleep Disorder" OR "Limit-Setting Sleep Disorders" OR ("Sleep Disorders" AND "Limit-Setting") OR ("Sleep Disorder" AND Limit-Setting) OR ("Sleep Disorder" AND "Limit Setting") OR "Nocturnal Eating-Drinking Syndrome" OR ("Eating-Drinking Syndrome" AND Nocturnal) OR ("Eating-Drinking Syndromes" AND Nocturnal) OR "Nocturnal Eating Drinking Syndrome" OR "Nocturnal Eating-Drinking Syndromes" OR (Syndrome AND "Nocturnal Eating-Drinking") OR (Syndromes AND "Nocturnal Eating-Drinking") OR "Adjustment Sleep Disorder" OR "Adjustment Sleep Disorders" OR ("Sleep Disorders" AND Adjustment) OR ("Sleep Disorder" AND Adjustment) OR "Environmental Sleep Disorder" OR "Environmental Sleep Disorders" AND ("Sleep Disorders" AND Environmental) OR ("Sleep Disorder" AND Environmental)) AND ("2019/11/01"[PubDate] : "2021/12/31"[PubDate]) |
| ProQuest | 2507 | February 19, 2021 | ("2019 nCoV" OR 2019nCoV OR "2019 novel coronavirus" OR "COVID 19" OR COVID19 OR "new coronavirus" OR "novel coronavirus" OR "SARS CoV-2" OR (Wuhan AND coronavirus) OR "COVID 19" OR "SARS-CoV" OR "2019-nCoV" OR "SARS-CoV-2") AND (insomnia OR "sleep problems" OR "Sleep quality" OR Dyssomnia OR "Sleep Disorders" OR "Extrinsic Sleep Disorder" OR "Extrinsic Sleep Disorders" OR "Limit-Setting Sleep Disorder" OR "Limit Setting Sleep Disorder" OR "Limit-Setting Sleep Disorders" OR "Nocturnal Eating-Drinking Syndrome" OR ("Eating-Drinking Syndrome" AND Nocturnal) OR ("Eating-Drinking Syndromes" AND Nocturnal) OR "Nocturnal Eating Drinking Syndrome" OR "Nocturnal Eating-Drinking Syndromes" OR (Syndrome AND "Nocturnal Eating-Drinking") OR (Syndromes AND "Nocturnal Eating-Drinking") OR "Adjustment Sleep Disorder" OR "Adjustment Sleep Disorders" OR ("Sleep Disorders" AND Adjustment) OR ("Sleep Disorder" AND Adjustment) OR "Environmental Sleep Disorder" OR "Environmental Sleep Disorders" AND ("Sleep Disorders" AND Environmental)) OR ("Sleep Disorder" AND Environmental) |
| Scopus | 2518 | February 19, 2021 | ("2019 nCoV" OR 2019ncov OR "2019 novel coronavirus" OR "COVID 19" OR covid19 OR "new coronavirus" OR "novel coronavirus" OR "SARS CoV-2" OR ( wuhan AND coronavirus ) OR "COVID 19" OR "SARS-CoV" OR "2019-nCoV" OR "SARS-CoV-2" ) AND ( insomnia OR "sleep problems" OR "Sleep quality" OR dyssomnia OR "Sleep Disorders" ) |
| Embase | 1426 | February 17, 2021 | ('2019 ncov' OR 2019ncov OR '2019 novel coronavirus'/exp OR '2019 novel coronavirus' OR covid19 OR 'new coronavirus' OR 'novel coronavirus' OR 'sars cov-2' OR (wuhan AND ('coronavirus'/exp OR coronavirus)) OR 'covid 19'/exp OR 'covid 19' OR 'sars-cov'/exp OR 'sars-cov' OR '2019-ncov' OR 'sars-cov-2') AND ('insomnia'/exp OR insomnia OR 'sleep problems' OR 'sleep quality'/exp OR 'sleep quality' OR 'dyssomnia'/exp OR dyssomnia OR 'sleep disorders'/exp OR 'sleep disorders') |
| ISI web of Knowledge | 474 | February 19, 2021 | (TS= "2019 nCoV" OR TS= “2019ncov” OR TS= "2019 novel coronavirus" OR TS="COVID 19" OR TS=covid19 OR TS="new coronavirus" OR TS= "novel coronavirus" OR TS="SARS CoV-2" OR TS= ( wuhan AND coronavirus ) OR TS="COVID 19" OR TS="SARS-CoV" OR TS="2019-nCoV" OR TS="SARS-CoV-2" ) AND (TS=insomnia OR TS="sleep problems" OR TS="Sleep quality" OR TS= dyssomnia OR TS="Sleep Disorders") |
| Total | 7263 |  |  |
| Full text assessment | 555 |  |  |
| Included | 177 |  |  |
